# The complete mitogenome of a 500-year-old Inca child mummy

**DOI:** 10.1038/srep16462

**Published:** 2015-11-12

**Authors:** Alberto Gómez-Carballa, Laura Catelli, Jacobo Pardo-Seco, Federico Martinón-Torres, Lutz Roewer, Carlos Vullo, Antonio Salas

**Affiliations:** 1 Unidade de Xenética, Departamento de Anatomía Patolóxica e Ciencias Forenses, and Instituto de Ciencias Forenses, Grupo de Medicina Xenómica (GMX), Facultade de Medicina, Universidade de Santiago de Compostela, 15872, Galicia, Spain; 2 Grupo de Investigación en Genética, Vacunas, Infecciones y Pediatría (GENVIP), Hospital Clínico Universitario and Universidade de Santiago de Compostela (USC), Galicia, Spain; 3 Translational Pediatrics and Infectious Diseases, Department of Pediatrics, Hospital Clínico Universitario de Santiago, Santiago de Compostela, Galicia, Spain; 4 Equipo Argentino de Antropología Forense, Independencia 644–3A, Edif. EME1, Córdoba, Argentina; 5 Institute of Legal Medicine and Forensic Sciences, Department of Forensic Genetics, Charité-Universitätsmedizin Berlin, Germany

## Abstract

In 1985, a frozen mummy was found in Cerro Aconcagua (Argentina). Archaeological studies identified the mummy as a seven-year-old Inca sacrifice victim who lived >500 years ago, at the time of the expansion of the Inca Empire towards the southern cone. The sequence of its entire mitogenome was obtained. After querying a large worldwide database of mitogenomes (>28,000) we found that the Inca haplotype belonged to a branch of haplogroup C1b (C1b*i*) that has not yet been identified in modern Native Americans. The expansion of C1b into the Americas, as estimated using 203 C1b mitogenomes, dates to the initial Paleoindian settlements (~18.3 thousand years ago [kya]); however, its internal variation differs between Mesoamerica and South America. By querying large databases of control region haplotypes (>150,000), we found only a few C1b*i* members in Peru and Bolivia (e.g. Aymaras), including one haplotype retrieved from ancient DNA of an individual belonging to the Wari Empire (Peruvian Andes). Overall, the results suggest that the profile of the mummy represents a very rare sub-clade that arose 14.3 (5–23.6) kya and could have been more frequent in the past. A Peruvian Inca origin for present-day C1b*i* haplotypes would satisfy both the genetic and paleo-anthropological findings.

In the summer of 1985 a group of mountaineers discovered a frozen mummy only partially unearthed at an altitude of 5,300 meters in the southwestern edge of the Aconcagua Mountain (“Cerro”), at the base of the Pirámide Mountain, in the Argentinean province of Mendoza. Instead of excavating the body, the mountaineers returned to their localities and informed specialists about this find. Excavations were subsequently carried out by professional archaeologists. In the ritual burial, archaeologists identified a very well preserved seven-year-old boy wrapped in numerous textiles and surrounded by six statuettes^1^. A range of archaeological and anthropological studies identified the mummy as the victim of an Inca sacrifice, a ritual known as “capacocha”, occurring approximately 500 years ago^1^. The ceremony (also known as “capac hucha”) involved the ritual sacrifice of children and it is interpreted today as one of several strategies used by the Inca state to integrate and control its vast civilization. The sacrificial rites involved children of great physical beauty and health in honor of the gods; the rituals were performed during or after important events (death of an emperor, the birth of a royal son, a victory in battle or an annual or biennial event in the Inca calendar), or in response to catastrophes (earthquakes, volcanic eruptions and epidemics)^2^,^3^. The children were selected from different locations throughout the Inca territories and taken to the high mountaintops for capacocha.

The Inca constituted the largest (about 12 million people) and one of the most complex civilizations in pre-Columbian America. Its political, administrative and military center was located in Cusco (modern-day Peru). The Inca arose in the highlands of Peru in the early 13^th^ century. From 1438 to 1533 they conquered or peacefully assimilated a large portion of western South America, including present-day Peru, a large part of Ecuador, south-central Bolivia, southern Colombia, northwest Argentina, and north-central/north Chile. The Aconcagua mummy dates to this period of expansion southwards, and was found close to the southernmost edge of the Inca expansion. There is abundant archaeological evidence supporting the practice of sacrifice within Inca society^4^. The last emperor of the Incas (Atahuallpa) was executed in 1533 by the soldiers of the Spanish conqueror Francisco Pizarro, marking the end of 300 years of Inca civilization.

The analysis of ancient DNA (aDNA) flourished in the last decade with the arrival of new sequencing technologies^5^,^6^,^7^,^8^. However, relatively few genetic studies have been carried out on mummies^9^,^10^,^11^,^12^,^13^,^14^,^15^. Ermini *et al.*^10^ analyzed the Tyrolean Iceman, a 46-year-old man who lived in the Neolithic-Copper Age transition in Central Europe about 5 kya; this represented the first complete mitochondrial genome sequence of a prehistoric European. To the best of our knowledge, there are only a few studies carried out on Native American mummies; all of them only targeted the mitochondrial DNA (mtDNA) first hypervariable region (HVS-I). For instance, Monsalve *et al.*^9^ analyzed the Kwäday Dän Ts’ìchi ancient remains of a man found in a melting glacier in British Columbia (Canada); the authors could characterize his mtDNA as belonging to haplogroup A, and found matches in populations from across the whole American continent. Wilson *et al.*^16^ analyzed samples from another Inca child sacrifice employing a multidisciplinary approach. These authors reported the mtDNA HVS-I and a few mtDNA SNPs from seven samples, allowing them to broadly allocate these haplotypes to Native American haplogroups C and D. The HVS-I segment of the remains of the mummy “Juanita” (also known as “Lady of Ampato” or “Inca Ice Maiden”) was published in GenBank (Acc. No. EF660742 and EF660743); this was identified as another Inca sacrifice victim: a 12–14-year-old child who lived in Mount Ampato (near Arequipa, Peru) about 500 years ago; its mtDNA haplotype could be allocated to haplogroup A (its sequence motif is widespread across the whole American continent).

The present study represents the first attempt at reporting the entire mtDNA of a Native American mummy, with the additional interest that this mummy represents a member of the Inca civilization. The main aim is to shed light on the genetic variation existing at the time of the Inca civilization and to interpret this variation in the light of the patterns observed in present-day populations.

## Results and Discussion

**Figure 1:**
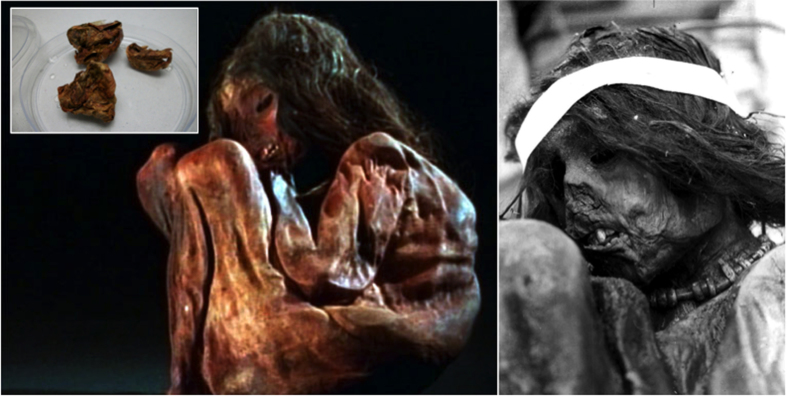
The Aconcagua mummy. The inset shows a picture of a portion of dissected lung from the mummy. A small piece of 350 mg was used for DNA extraction. The photo of the mummy has been taken from^49^ and it is reproduced here with the permission of the University of Cuyo Publisher (Argentina).

DNA was extracted from a small piece of lung of the mummy (Fig. 1). The haplotype of the mummy has 51 variants with respect to the rCRS^17^ (Table 1). The variation observed in the mummy’s mitogenome fits perfectly within the phylogeny of one of the most typical Native American haplogroups, C1b. Moreover, this haplogroup represents one of the most frequent branches within C1 (together with C1c and C1d) and was recently identified as one in more than fifteen mtDNA American founders^18^,^19^,^20^,^21^.

**Figure 2:**
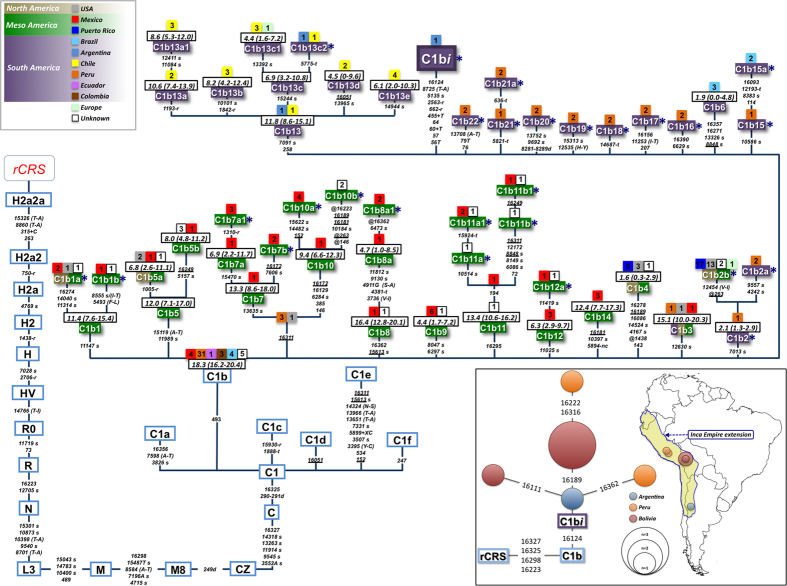
Skeleton of the global C1b phylogeny. The C1b1*i* clade represented by the haplotype found in the Inca mummy is also located in the phylogeny. There is one mitogenome (JX413043) that belongs to haplogroup C1b13b sampled in a Spanish individual, although born in Talagante (Chile); therefore we labeled it here as originating in America. TMRCA are indicated above haplogroup labels. An asterisk to the right of the haplogroup labels identifies sub-clades that were newly identified in this study, compared to the last version of Phylotree (Build 16). The position of the revised Cambridge reference sequence (rCRS) is indicated for reading sequence motifs^17^. Mitochondrial DNA variants are indicated along the branches of the phylogenetic tree. Mutations are transitions unless a suffix A, C, G, or T indicates a transversion. Other suffixes indicate insertions (+), synonymous substitution (s), mutational changes in tRNA (−t), mutational changes in rRNA (−r), noncoding variant located in the mtDNA coding region (−nc) and an amino acid replacement (indicated in round brackets). Variants underlined represent recurrent mutations in this tree, while a prefix ‘@’ indicates a back mutation. Mutational hotspot variants at positions 16182, 16183, and 16519, as well as variation around position 310 and length or point heteroplasmies were not considered for the phylogenetic reconstruction. The numbers in small squares attached above haplogroup labels indicate the number of occurrences (mitogenomes) of the corresponding haplogroups available in the public domain (literature and/or GenBank); the color of the squares indicates their geographic origin according to the legend inset. More details on the geographic or ethnic origin of all the mitogenomes used in this network are provided in Supplemental Data Table S1. The bottom-right inset shows a network of HVS-I sequences that potentially belong to haplogroup C1b*i* (left) and a map of South America showing their geographic location (right). The map was built using a blank map based on GPS coordinates and the SAGA v. 2.1.1 (http://www.saga-gis.org/; see methods).

A total of 201 C1b mitogenomes could be compiled from the literature (although two of them lacking information on the control region). The phylogeny of C1b was reconstructed and updated (see full phylogeny in Figure S1 and its skeleton in Fig. 2) using the latest version available in Phylotree Build 16 (http://www.phylotree.org), which allowed dating the Time of the Most Recent Common Ancestor (TMRCA) at different C1b ancestral nodes. In this phylogenetic analysis, 23 new minor sub-clades could be identified, all of them characterized by at least two different mitogenomes. C1b is approximately 18.3 (16.2–20.4) kya (Table 2; Fig. 2), thus supporting previous assessments indicating that C1b most likely arose relatively early, either in Beringia or at a very initial stage of the Paleoindian southward migration^19^,^22^. In agreement with previous findings^18^,^23^, the fact that C1b is only slightly older in Mesoamerica than in South America (Table 2) confirms that the southward expansion of this clade was very rapid^18^. While some C1b sub-clades were exclusively observed in Mesoamerica or in South America, a few of them were found in both territories.

**Figure 3:**
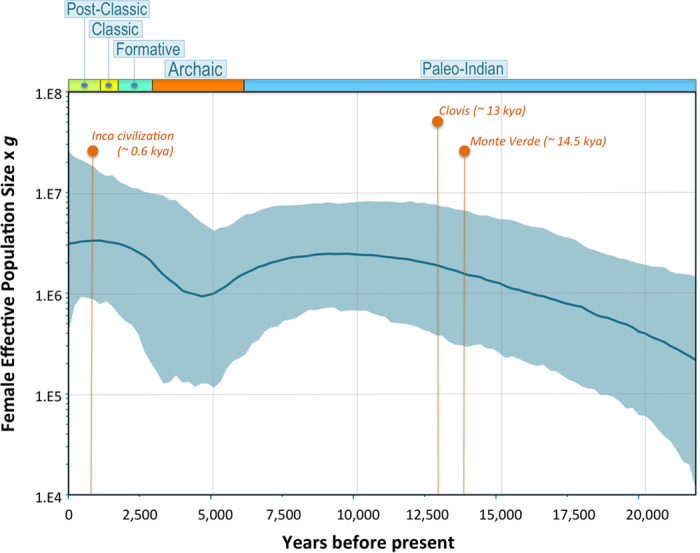
BSP indicating the median of the hypothetical effective population size through time based on data from the C1b mitogenomes. The maximum time is the median posterior estimate of the genealogy root-height.

The BSP of C1b (Fig. 3) overall points to one major episode of constant population growth within America that starts very early during the initial Paleo-Indian settlement into the American continent and their spread southwards. Approximately 9 kya there is a progressive decrease of effective population size that last until about 5 kya. Then the BSP indicates a new episode of constant population growth broadly coinciding with the beginning of the Archaic period, that is, with the increasingly intensive gathering of a wide range of resources and the decline of the hunting lifestyle. This progressive growth expands during the Formative and the Classic period (therefore including the initial periods of development of the Inca civilization). Only at very recent times, the BSP seems to suggest a final episode of population reduction, fitting with the arrival of Europeans.

**Figure 4:**
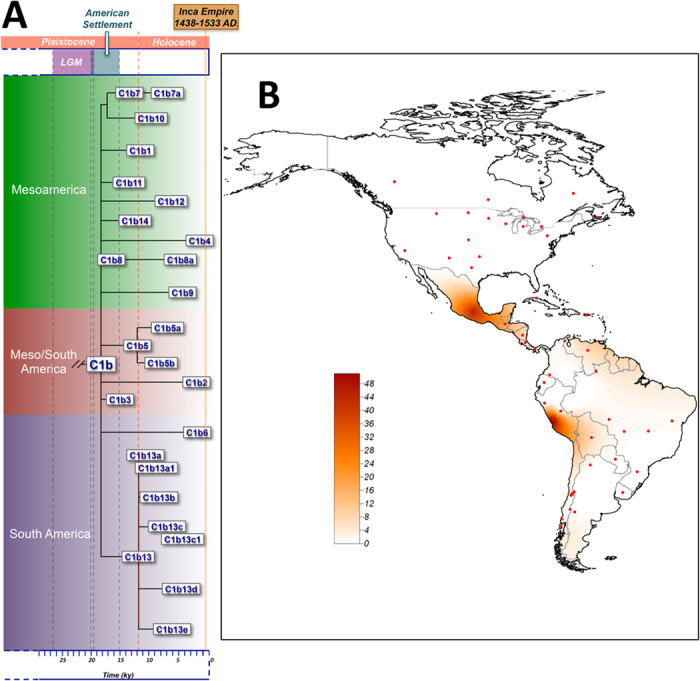
(**A**) ML phylogeny and TMRCA of the main C1b clades analyzed in the present study and the American chronology (LGM: Last Glacial Maximum). (**B**) Spatial-frequency distribution of haplogroup C1b. The map was built as indicated in Figure 2 and based on control region information. Note there are two main peaks of haplogroup C1b frequencies, one centered in Mexico and another one in Peru. In addition, there is a third peak in Puerto Rico (*n* = 23); this high frequency on the island projects over the North-East of South America (i.e. Venezuela and North of Brazil) where in reality C1b is virtually absent. The map was built using a blank map based on GPS coordinates and the SAGA v. 2.1.1 (see methods).

Figure 4 shows that C1b mitogenomes were found around two main geographic locations, one in Mesoamerica, and the other one around Peru and extending southwards from here along the Pacific coast. Figure 4 also shows a phylogenetic skeleton of the main C1b branches in the American chronology context. The observed pattern of geographic variation is compatible with the following broad migration scenario for C1b carriers: (*i*) early and rapid spread of C1b across the full American continent during the period of initial continental settlement; (*ii*) important population isolation of the Mesoamerican and the South American gene pools for long periods, as indicated by the presence of very old and very young clades exclusively found in each of these two sub-continental regions; and (*iii*) sporadic gene exchange between both sub-continental regions, as suggested by the existence of a few clades that are present today in Mesoamerica and South America; these clades have ages ranging from 15 kya (C1b3) to 2 kya (C1b2) ago. The main ancient American civilizations, such as the Maya and the Inca, could have contributed to the gene flow between the main continental regions^24^.

The haplotype of the Inca mummy belongs to a new clade that branches off from the root of haplogroup C1b, thus providing additional support for the authenticity of the haplotype (see M&M section). This new clade, C1b*i* (where ”i” stands for ‘Inca’), has 10 private mutations (Table 1). All private mutations were checked very carefully and replicated in confirmatory sequence analysis. Although there is no way to date C1b*i* using only one mitogenome, the amount of variation accumulated in the mummy’s haplotype is compatible with an old age, at least as old as other old branches within C1b that have accumulated a similar amount of variation. Figure S2 shows the number of mutations accumulated from the root of haplogroup C1b to all the tips of the phylogeny (Figure S1) and their relative frequency; the ten mutations observed in the mummy’s branch fall within expected values. The HVS-1 motif (Table 3) was used to search for members of C1b*i* in public haplotype databases (>170,000 partial mtDNA sequences). Only four samples belonging to C1b share the transition at position 16124 (Table 3; Fig. 2). A tentative dating can be carried out using these few control region haplotypes that fall within C1b*i* (Table 3). The TMRCA estimated from these haplotypes (based on *ρ*) is 14.3 (5.0-24.0) kya, which is consistent with the suggestion that C1b*i* constitutes an old clade. At the same time, the phylogeographic patterns of C1b*i* control region haplotypes point to a distribution of this lineage constrained to South America. Moreover, these patterns fit well with the maximum extension of the Inca Empire around 1525, when the mandate of Huayna Capac (eleventh “*Sapa”* of the Inca Empire) ended. Within C1b, there is a different sub-clade that shows very similar characteristics to C1b*i*, namely, C1b13. The TMRCA of this sub-clade is 11.8 (8.6–15.1) kya; it is virtually absent from North-Central America and its geographic location is mainly centered in Chile. C1b13 most likely arose in the Southern Cone region and differentiated locally soon after human arrival, during the tribalization and linguistic differentiation process^25^. The geographic distribution of C1b13 also fits well with the expansion of the Inca Civilization into the northern territories of Chile although its age is much older, thus suggesting that perhaps only some (still un-sampled) sub-lineages of C1b*i* might be related to the Inca’s timeline (as it occurs with other C1b sub-clades; Fig. 4).

In conclusion, the phylogenetic patterns of C1b*i* point to a geographic origin in the Andean side of the South American sub-continent approximately 14 kya. The haplotype found in the Inca child from the Cerro Aconcagua, interpreted in the light of present-day variation in South America and together with the different archaeological and anthropological findings, supports the existence of demographic movements along the Pacific coastline during the Inca period. The fact that C1b*i* is very uncommon in present-day populations from South America could be explained by insufficient sampling of modern populations (although the present-day haplotype databases of mitogenomes and partial mtDNA sequences are very large). Alternatively, this rarity could reflect important changes in the gene pool of South America since the period of the Inca civilization. Further research on modern and ancient South American populations, preferably based on the sequencing of mitogenomes to a population level, would probably allow a better understanding of the maternal lineage observed in the mummy and the demography of the Incas.

## Material and Methods

### Mummy DNA extraction and quantification

A dissected lung from the Aconcagua’s child was used for DNA extraction (Fig. 1). The post-mortem time was approximately 500 years according to the anthropological and historical information obtained^1^. The lung was preserved at −20° C since it was exhumed in 1985.

All DNA protocols were carried out in dedicated laboratories especially designed for DNA extraction of aged samples with facilities to minimize the risk of contamination, following ISFG recommendations^26^. Operators used the necessary equipment to avoid contamination of the samples at every step of the analysis (full-body sterile suit, gloves, face screen, etc). Plastic-ware used was DNA-free, autoclaved and UV irradiated as an additional precaution. All steps were carried out in laminar flow cabinets. Reagent blanks accompanying the extraction procedure were processed and checked for contamination.

Using a sterile scalpel and forceps, an inner piece of the lung’s tissue sample was extracted and further placed inside a sterile disposable Petri dish. All outer surfaces were discarded to prevent contamination from contemporary DNA. Thus, a 350 mg piece of lung tissue, brown colored and showing signs of dehydration was obtained and placed inside a 50 mL sterile tube. Details on the extraction protocol are provided in Supplemental data Text S1.

The DNA extract was quantified using the AB QuantifilerTM Human DNA Quantification kit with the 7500 real-time PCR system according to the manufacturer’s protocol. Based on the quantification results, the sample was diluted for mtDNA control region PCR amplification.

### Mitochondrial DNA amplification and sequencing

Sequencing of the mummy mtDNA control region was carried out in the two laboratories involved in the present study. All PCR reactions included a negative control as well as 9947A control DNA. A minimum of two amplifications were performed on the DNA extract. Replicate analysis of the same extracts was carried out to provide duplicated control region sequence consensus. There was no evidence of contamination in any reagent blanks or negative PCR controls (Figure S3).

For amplification and sequencing reactions the following primers pairs for the control region were used: 15967F-16429R, 16268F-186R, 6F-430R, 350F-639R in the Argentinean lab and 15997F-16401R, 16380F-17R, 16555F-408H, 332F-599H in the Spanish lab. The complete genome was sequenced using the primers described by Kivisild *et al.*^27^ and following protocols previously described^28^. PCR conditions were previously described^29^,^30^.

All PCR products obtained were checked by agarose gel electrophoresis. For purification of amplified PCR, products EXOSAPit was used.

Sequencing reactions were performed by using BDTv3.1 sequencing kit (Applied Biosystems) using 3 μl of PCR purified product template. Centrisep columns and EdgeBio Performa® DTR V3 96-Well plates were the sequencing purification method. Capillary electrophoresis was performed in an ABI Prims 3130 in the Argentinean lab and ABI 3730 Genetic Analyzer (Life Technologies, CA, USA) in the Spanish lab. Sequencing Analysis 5.2 software was used for quality sequencing analysis. Sequence edition was performed by using Sequencher (Ann Arbor, MI, USA) in the Argentinean lab and SeqScape (AB) in the Spanish lab; the sequences obtained were analyzed against the rCRS (Revised Cambridge Reference Sequence). *A posteriori* sequence quality was evaluated following the methods described earlier^31^,^32^.

The mtDNA control region was obtained by two independent laboratories from Argentina (Córdoba) and Spain (Santiago de Compostela). A blind test comparison of the sequencing results was carried out: the results were cross-compared and matched completely. The DNA of the mummy was well preserved as indirectly indicated by the good quality of the sequencing results obtained in both laboratories.

The mtDNA of all lab operators was sequenced in order to facilitate the identification of potential contamination from biological sources.

Table 1 shows the full variation observed in the mummy’s haplotype. Note that the alignment of the haplotype found in the mummy shows length variability in the HVS-II segment, and this alignment allows for several nomenclatures such as (*i*) 56 T 56 + C and (*ii*) 56 T 57 60 + T. Although the first option seems more parsimonious, the second option fits better with the global phylogeny^26^.

### Consideration of contamination issues

Previous studies on ancient DNA assumed that a certain amount of contamination is unavoidable even in ancient specimens that were excavated following dedicated protocols that pay special attention to the contamination issue^10^,^33^. It is also well-known that the impact of contamination is highly dependent on the degree of preservation of the original DNA^34^. The case of the Inca mummy is different from most previous studies on ancient DNA, and it bears some similarities with the Tyrolean Iceman^10^ in terms of preservation with the additional advantage of the Inca mummy being much younger than the Tyrolean Iceman. Thus, the specimen analyzed in the present study is a mummified human body that underwent a spontaneous freeze-desiccation process and was preserved at a very low temperature. Several lines of evidence suggested a very good preservation of the mummy’s endogenous DNA:

- The mummy was almost completely buried at the time of its discovery. It was moved directly to a freezer and remained frozen all the time until DNA extraction. Extraction of lung tissue was done in an operating room in completely sterile conditions. In order to further prevent potential contamination, an inner portion of the lung piece was taken for DNA extraction in a dedicated laboratory.
- Its entire mtDNA control region haplotype was obtained by two independent laboratories. The results were cross-compared and matched completely.
- Mitochondrial DNA of all the operators was obtained and cross-checked with the haplotype of the mummy (Supplemental Data Table S2). These haplotypes were phylogenetically incompatible with the same haplogroup observed in the mummy’s haplotype.
- The variation observed in the mummy fits perfectly with the expected Native American phylogeny, and therefore there is no indication of sequence artifacts^31^,^35^. Moreover, the phylogenetic characteristics of private mutations observed do not consistently point to the presence of contaminant haplotypes and artificial recombination^36^,^37^,^38^.
- Multiple heteroplasmies are not common and would often point to sequencing problems^31^. No point heteroplasmies were detected in the mummy’s sequences.
- The mummy’s haplotype has 10 mutations on top of the of the C1b root. The fact that this haplotype identifies a new lineage that does not fall within the main extant sub-clades of the C1b and the global Native American phylogeny (represented by 201 and >1,200 complete mitogenomes, respectively), adds additional support to the authenticity of the sequence^10^. There are only a few control region haplotypes that fit with the mummy’s haplotype motif (*n* = 6 from an American database of more than 170,000 haplotypes), indicating that this haplogroup is at least very rare in present-day populations and, therefore, very unlikely to appear as contaminant in our analysis^10^.
- The 10 mutations that characterize C1bi are private within C1b haplogroup, but only the transition T662C has not been observed previously in databases and internet searches (executed as in^39^) carried out on worldwide mtDNA variation.
- The amount of variants accumulated in this haplotype with respect to what is observed for the many C1b clades also falls within the expected range as can be estimated from the phylogeny (Supplemental Data Figure S2).

### Statistical analysis

Maximum parsimony trees were built for C1b mitogenomes. Figure 2 shows the skeleton of C1b phylogeny. TMRCA of this mitogenome phylogeny was computed using of a maximum likelihood (ML) procedure according to Phylotree 16 phylogeny (Table 2). For this purpose, the software PAML 4.4^40^ was used assuming the HKY85 mutation model (ignoring indels, as per common practice) and using gamma-distributed rates (approximated by a discrete distribution with 32 categories) and three partitions: HVS-I (positions 16051–16400), HVS-II (positions 68–263), and the remainder. The TMRCA of entire mitogenomes and C1b*i* control region sequences (Fig. 2) was also computed using the averaged distance (*ρ*) of all the haplotypes in a clade to the respective root haplotype. This was done (*i*) using the whole variation observed in mitogenomes, and (*ii*) considering only synonymous mutations. An heuristic estimate of the standard error (σ) was calculated from an estimate of the genealogy^41^. All TMRCA calculations were obtained using the entire mtDNA genomes but excluding hotspot mutations such as 16182C, 16183C and 16519. The ‘star-likeness’ of the trees was measured using the star index *ρ*/*n*×*σ*^2^; this index can take values between 1/*n* (single haplotype representing *n* mtDNAs) and 1 (perfect star phylogeny)^19^,^42^.

Both methods, ML and *ρ*, yielded very similar divergence ages (Table 2). There were anomalous behaviors on dates obtained for some sub-clades (e.g. C1b1, C1b3; Table 2); which could be explained by the under/overrepresentation of some sub-clade in the phylogeny (in a similar way as it was observed for haplogroup A2w1 in Söchtig *et al.*^24^) and/or because the star-likeness of some branches is very low (C1b1, C1b2, etc). Moreover, ages computed using *ρ* make no phylogenetic sense for some sub-haplogroups; for instance the age of C1b1 using synonymous mutations is older than the age of the whole C1b haplogroup. Overall, ages obtained using ML seem to be more consistent and these were the ones used for discussion throughout the text with the exception of coalescent ages computed on control region for which only *ρ* estimates were obtained.

Mutational distances were converted into years using the corrected evolutionary rate (and the calculator) from Soares *et al.*^43^, namely, for the entire molecule (1.16649 ×10^−8^ substitutions per nucleotide per year or one mutation every 3624 years) and for the control region (HVS-I: 1.64273 ×10^−7^; HVS-II: 2.2964 ×10^−7^). Standard deviations of age estimates are noted as SD throughout the text.

Bayesian skyline plots^44^ (BSPs) were obtained using the software BEAST v1.8^45^ for the complete C1b sequences using (*i*) a relaxed molecular clock (lognormal in distribution across branches and uncorrelated between them)^46^, and (*ii*) the HKY model of nucleotide substitutions with gamma-distributed rates. Ancestral gene trees for each region were inferred using a GTR substitution model. Each MCMC sample was based on a run of 100 million generations, with samples drawn every 20,000 MCMC steps, after a discarded burn-in of 10,000,000 steps. BSPs were visualized using Tracer v.1.6^47^. We used a strict molecular clock with a mutation rate of 1.16649 ×10^−8^ substitution/site/year for the entire mitogenome^43^. We used a generation time of 25 years, as in Fagundes *et al.*^48^.

Phylogeographic searches of mtDNA haplotypes were carried out in an in-house database containing >27,000 mitogenomes, and >170,000 partial (mainly HVS-I) mtDNA sequences. Additional searches were carried out on EMPOP (http://empop.org), Familytree (https://familysearch.org/), Mitosearch (http://www.mitosearch.org), and the Sorenson (http://www.smgf.org/) databases.

The geographic representation of C1b mitogenomes was carried out using SAGA v. 2.1.1 (http://www.saga-gis.org/). We used the ordinary Kriging method for interpolating haplogroup frequencies.

## Additional Information

**How to cite this article**: Gómez-Carballa, A. *et al.* The complete mitogenome of a 500-year-old Inca child mummy. *Sci. Rep.* **5**, 16462; doi: 10.1038/srep16462 (2015).

## Supplementary Material

Supplementary Information

Supplementary Information

## Figures and Tables

**Table 1:** Variants found in the mitogenome of the Inca mummy using the rCRS^17^ as a reference.

Variant	Substitution	Location	Aminoacidchange	Type
56 (P)	A-T	HVS-2	−	R14
57 (P)	T-C	HVS-2	−	H15–T2b3a1–W5a1a1a
60 + T (P)	−	HVS-2	−	R0a2'3–R04–W5a1a1a
64 (P)	C-T	HVS-2	−	L0a2–M15–M27b–M74a–M52b1a–A8a–S3–X2n–R0a’b–H1b1g–H57–F1a1b–F2e1–K1a19a
73	A-G	HVS-2	−	R0–R
249d	−	HVS-2	−	M8–CZ
263	A-G	HVS-2	−	H2a2a–H2a2
290–291d	−	HVS-2	−	C–C1
309 + C	−	HVS-2	−	Hotspot
315 + C	−	HVS-2	−	Hotspot
455 + T (P)	−	HVS-2	−	L5a–M5b2b1–M7c1c3i–I1–N1b1a1–B4c1b1–U1a2
489	T-C	HVS-2	−	L3–M
493	A-G	HVS-2	−	C1–C1b
523–524d	−	HVS-2	−	Hotspot
662 (P)	T-C	12 S	−	–
750	A-G	12 S	−	H2a2–H2a
1438	A-G	12 S	−	H2–H
2563 (P)	T-C	16 S	−	–
2706	A-G	16 S	−	H2–HV
3552	T-A	ND1	−	CZ–C
4715	A-G	ND2	−	M–M8
4769	A-G	ND2	−	H2a–H2
5135 (P)	C-T	ND2	−	N7b
7028	C-T	CO1	−	H2–HV
7196	C-A	CO1	−	M–M8
8584	G-A	ATP6	A-T	M–M8
8701	A-G	ATP6	T-A	N–L3
8725 (P)	A-G	ATP6	T-A	L0d2c2
8860	A-G	ATP6	T-A	H2a2a–H2a2
9540	T-C	CO3	−	N–L3
9545	A-G	CO3	−	CZ–C
10398	A-G	ND3	T-A	N–L3
10400	C-T	ND3	−	L3–M
10873	T-C	ND4	−	N–L3
11719	G-A	ND4	−	R0–R
11914	G-A	ND4	−	CZ–C
12705	C-T	ND5	−	R–N
13263	A-G	ND5	−	CZ–C
14318	T-C	ND6	N-S	CZ–C
14766	C-T	CYTB	T-I	HV–R0
14783	T-C	CYTB	−	L3–M
15043	G-A	CYTB	−	L3–M
15301	G-A	CYTB	−	N–L3
15326	A-G	CYTB	T-A	H2a2a–H2a2
15487	A-T	CYTB	−	M–M8
16124 (P)	T-C	HVS-1	−	L3b–L3d–M3c1b1–C4b2–M69a–H2a1e1a–H5t–R9b1b
16223	C-T	HVS-1	−	R–N
16298	T-C	HVS-1	−	M–M8
16325	T-C	HVS-1	−	C–C1
16327	C-T	HVS-1	−	CZ–C
16519	T-C	HVS-1	−	Hotspot

Private variants within the C1b phylogeny are indicated as (P) in the first column. The “Type” column indicates whether this variant had been detected previously in other haplogroup contexts within the worldwide phylogeny (according to Phylotree Build 16), or whether it is a hotspot (thus present in a large number of different branches of the mtDNA phylogeny). Transition T2563C is not in the skeleton of Phylotree Build 16 but it appeared sporadically in some mitogenome (e.g. HQ713446 [haplogroup A4]).

**Table 2:** Haplogroup coalescence time estimates in kya for C1b and its sub-clades based on ML and the computation of the averaged distance (AD) of the haplotypes of a given clade to the respective root haplotype.

*Haplogroup*	*n*	*ML*		AD					AD (only synonymous mutations)				
		*TMRCA*	95% CI	TMRCA	95% CI	ρ	σ	ρ/(n×σ2)	TMRCA	95% CI	ρ	σ	ρ/(n×σ2)
C1b	200	18.29	16.22−20.39	17.27	14.1–26.63	6.39	0.50	0.1	19.67	16.86–22.48	2.51	0.36	0.1
C1b + 16311	18	17.10	14.39−19.84	18.85	12.55–25.35	6.94	1.14	0.3	21.02	15.28–26.77	2.67	0.73	0.3
C1b7	7	13.30	8.65−18.06	11.39	5.93–17.03	4.29	1.03	0.6	11.26	5.75–16.78	1.43	0.70	0.4
C1b7a	4	6.92	2.20−11.77	7.89	2.38–13.59	3.00	1.06	0.7	1.97	0.00–3.94	0.25	0.25	1.0
C1b10	7	9.44	6.60−12.33	12.97	5.35–20.09	4.86	1.43	0.3	14.64	7.61–21.68	1.86	0.89	0.3
C1b1	5	11.42	7.56−15.37	11.16	4.08–18.53	4.20	1.34	0.5	20.5	12.01–28.99	2.60	1.08	0.4
C1b11	9	13.43	10.65−16.25	13.68	6,16–21.5	5.11	1.41	0.3	14.02	8.07–19.96	1.78	0.75	0.4
C1b12	5	6.29	2.91−9.75	5.22	1.37–9.16	2.00	0.75	0.7	4.73	1.2–8.26	0.60	0.45	0.6
C1b13	22	11.81	8.56−15.12	11.48	7.99–15.05	4.32	0.65	0.5	12.18	8.57–15.8	1.55	0.46	0.3
C1b13a	5	10.59	7.36−13.88	15.61	8.2–23.31	5.80	1.37	0.6	12.61	6.71–18.51	1.60	0.75	0.6
C1b13a1	3	8.62	5.30−12.00	15.23	7.82–22.94	5.67	1.37	1.0	13.14	7.26–19.02	1.67	0.75	1.0
C1b13b	3	8.24	4.21−12.37	9.7	3.89–15.7	3.67	1.11	1.0	10.51	5.26–15.77	1.33	0.67	1.0
C1b13c	6	6.92	3.16−10.77	6.55	1-76-11.49	2.50	0.93	0.5	6.57	1.15–11.99	0.83	0.69	0.3
C1b13c1	4	4.37	1.62−7.18	4.55	0.72–8.49	1.75	0.75	0.8	1.97	0.00–3.94	0.25	0.25	1.0
C1b13d	2	4.50	0.00−9.65	3.9	0.00–8.42	1.50	0.87	1.0	3.94	0.00–7.88	0.50	0.50	1.0
C1b13e	4	6.11	2.01−10.31	5.22	1.58–8.94	2.00	0.71	1.0	–	–	–	–	–
C1b14	3	12.46	7.71−17.33	14.3	7.14–21.74	5.33	1.33	1.0	10.51	5.26–15.77	1.33	0.67	1.0
C1b2	32	2.11	1.28−2.94	12.18	2.23–22.71	4.57	1.89	0.0	17.8	6.94–28.65	2.26	1.38	0.0
C1b3	3	15.12	10.05−20.33	14.3	7.14-21-74	5.33	1.33	1.0	5.26	1.54–8.97	0.67	0.47	1.0
C1b4	10	1.56	0.27−2.87	1.55	0.12–2.99	0.60	0.28	0.8	1.58	0.46–2.69	0.20	0.14	1.0
C1b5	8	12.00	7.14−17.00	11.64	5.67–17.81	4.37	1.12	0.4	15.77	10.02–21.51	2.00	0.73	0.5
C1b5a	4	6.79	2.56−11.14	6.55	2.46–10.75	2.50	0.79	1.0	7.88	3.94–11.83	1.00	0.50	1.0
C1b5b	4	7.99	4.81−11.23	8.57	2.32–15.05	3.25	1.20	0.6	15.77	8.39–23.14	2.00	0.94	0.6
C1b6	3	1.93	0.00−4.81	1.72	0.00–4.13	0.67	0.47	1.0	2.63	0.00–5.26	0.33	0.33	1.0
C1b8	5	16.44	12.85−20.09	22.47	11.88–33.59	8.20	1.91	0.4	20.5	11.72–29.28	2.60	1.11	0.4
C1b8a	3	4.68	0.97−8.48	7	1.04–13.18	2.67	1.15	0.7	7.88	2.01–13.76	1.00	0.75	0.6
C1b9	7	4.43	1.68−7.24	5.98	0.99–11.12	2.29	0.97	0.3	4.51	2.25–6.76	0.57	0.29	1.0

We used all the mitogenomes from Table S1. Estimates were additionally obtained for the complete genomes and considering only the synonymous variants. The summand *n* refers to the complete mtDNA sequences considered in each clade. Computation of TMRCA of C1b13 includes one European individual of South American descent.

**Table 3:** Haplotypes that are closely related to, or belong to, C1b*i* clade.

Ref.	*n*	Population	Region/Archeological site	Country(Province)	Variants(from C1b root)	Sequencerange
This study	1	Inca (AD 1480)	Cerro Aconcagua	Argentina (Mendoza)	16124	16024–16569
[1]	1	Aymara	Andahuayalas	Peru (Apurimac)	16124 16362	16024–16383
[2]	1	Post-Wari (AD 1000–1450)	Huari	Peru (Ayacucho)	16124 16189 16222 16316	16011–16382
[3]	3	Aymara	−	Bolivia (La Paz)	16124 16183C 16189	16024–16383
[4]	1	Bolivian	Pucarani	Bolivia (Cochabamba)	16111 16124	16024–16569

Ref.: [1]: Barbieri *et al.*^50^. [2]: Kemp *et al.*^15^. [3]: Batai *et al.*^51^. [4]: Taboada-Echalar *et al.*^52^.

## References

[b1] Schobinger J., Los santuarios de altura incaicos y el Aconcagua: aspectos generales e interpretativos. (Relaciones de la Sociedad Argentina de Antropología XXIV, Buenos Aires, Argentina, 1999).

[b2] Ceruti C. Human bodies as objects of dedication at Inca mountain shrines (north-western Argentina). World Archaeol 36(**1**), 103 (2004).

[b3] Andrushkom V. A. *et al.* Investigating a child sacrifice event from the Inca heartland. J Archaeol Sci 38(**2**), 323 (2011).

[b4] Wilson A. S. *et al.* Archaeological, radiological, and biological evidence offer insight into Inca child sacrifice. Proc Natl Acad Sci USA 110(**33**), 13322 (2013). 23898165 10.1073/pnas.1305117110 PMC3746857

[b5] Lazaridis I. *et al.* Ancient human genomes suggest three ancestral populations for present-day Europeans. Nature 513(**7518**), 409 (2014). 25230663 10.1038/nature13673 PMC4170574

[b6] Schroeder H. *et al.* Genome-wide ancestry of 17th-century enslaved Africans from the Caribbean. Proc Natl Acad Sci USA 112(**12**), 3669 (2015). 25755263 10.1073/pnas.1421784112 PMC4378422

[b7] Olalde I. *et al.* Derived immune and ancestral pigmentation alleles in a 7,000-year-old Mesolithic European. Nature 507(**7491**), 225 (2014). 24463515 10.1038/nature12960 PMC4269527

[b8] Green R. E. *et al.* A draft sequence of the Neandertal genome. Science 328(**5979**), 710 (2010). 20448178 10.1126/science.1188021 PMC5100745

[b9] Monsalve M. V. *et al.* Brief communication: molecular analysis of the Kwaday Dan Ts’finchi ancient remains found in a glacier in Canada. Am J Phys Anthropol 119(**3**), 288 (2002). 12365041 10.1002/ajpa.10116

[b10] Ermini L. *et al.* Complete mitochondrial genome sequence of the Tyrolean Iceman. Curr Biol 18(**21**), 1687 (2008). 18976917 10.1016/j.cub.2008.09.028

[b11] Kuch M. *et al.* A preliminary analysis of the DNA and diet of the extinct Beothuk: a systematic approach to ancient human DNA. Am J Phys Anthropol 132(**4**), 594 (2007). 17205549 10.1002/ajpa.20536

[b12] Hawass Z. *et al.* Revisiting the harem conspiracy and death of Ramesses III: anthropological, forensic, radiological, and genetic study. BMJ 345, e8268 (2012). 23247979 10.1136/bmj.e8268

[b13] Khairat R. *et al.* First insights into the metagenome of Egyptian mummies using next-generation sequencing. Journal of applied genetics 54(**3**), 309 (2013). 23553074 10.1007/s13353-013-0145-1

[b14] Kemp B. M. *et al.* Genetic analysis of early holocene skeletal remains from Alaska and its implications for the settlement of the Americas. Am J Phys Anthropol 132(**4**), 605 (2007). 17243155 10.1002/ajpa.20543

[b15] Kemp B. M., Tung T. A. & Summar M. L. Genetic continuity after the collapse of the Wari empire: mitochondrial DNA profiles from Wari and post-Wari populations in the ancient Andes. Am J Phys Anthropol 140(**1**), 80 (2009). 19294741 10.1002/ajpa.21037

[b16] Wilson A. S. *et al.* Stable isotope and DNA evidence for ritual sequences in Inca child sacrifice. Proc Natl Acad Sci USA 104(**42**), 16456 (2007). 17923675 10.1073/pnas.0704276104 PMC2034262

[b17] Andrews R. M. *et al.* Reanalysis and revision of the Cambridge reference sequence for human mitochondrial DNA. Nat. Genet. 23(**2**), 147 (1999). 10508508 10.1038/13779

[b18] Bodner M. *et al.* Rapid coastal spread of First Americans: Novel insights from South America’s Southern Cone mitochondrial genomes. Genome Res 22(**5**), 811 (2012). 22333566 10.1101/gr.131722.111 PMC3337427

[b19] Achilli A. *et al.* The phylogeny of the four pan-American MtDNA haplogroups: implications for evolutionary and disease studies. PLoS One 3(**3**), e1764 (2008). 18335039 10.1371/journal.pone.0001764 PMC2258150

[b20] Tamm E. *et al.* Beringian standstill and spread of Native American founders. PLoS ONE 2(**9**), e829 (2007). 17786201 10.1371/journal.pone.0000829 PMC1952074

[b21] Perego U. A. *et al.* The initial peopling of the Americas: a growing number of founding mitochondrial genomes from Beringia. Genome Res 20(**9**), 1174 (2010). 20587512 10.1101/gr.109231.110 PMC2928495

[b22] Reich D. *et al.* Reconstructing Native American population history. Nature 488(**7411**), 370 (2012). 22801491 10.1038/nature11258 PMC3615710

[b23] Perego U. A. *et al.* Distinctive Paleo-Indian migration routes from Beringia marked by two rare mtDNA haplogroups. Curr. Biol. 19(**1**), 1 (2009). 19135370 10.1016/j.cub.2008.11.058

[b24] Söchtig J. *et al.* Genomic insights on the ethno-history of the Maya and the ‘Ladinos’ from Guatemala. BMC Genomics 16(**1**), 131 (2015). 25887241 10.1186/s12864-015-1339-1 PMC4422311

[b25] de Saint Pierre M. *et al.* Arrival of paleo-indians to the southern cone of South america: new clues from mitogenomes. PLoS One 7(**12**), e51311 (2012). 23240014 10.1371/journal.pone.0051311 PMC3519775

[b26] Parson W. *et al.* DNA Commission of the International Society for Forensic Genetics: Revised and extended guidelines for mitochondrial DNA typing. Forensic Sci. Int. Genet. 13C, 134 (2014). 25117402 10.1016/j.fsigen.2014.07.010

[b27] Kivisild T. *et al.* The role of selection in the evolution of human mitochondrial genomes. Genetics 172(**1**), 373 (2006). 16172508 10.1534/genetics.105.043901 PMC1456165

[b28] Brisighelli F. *et al.* The Etruscan timeline: A recent Anatolian connection. Eur J. Hum. Genet. 17(**5**), 693 (2009). 19050723 10.1038/ejhg.2008.224 PMC2986270

[b29] Cerezo M. *et al.* High mitochondrial DNA stability in B-cell chronic lymphocytic leukemia. PLoS One 4(**11**), e7902 (2009). 19924307 10.1371/journal.pone.0007902 PMC2775629

[b30] Cerezo M. *et al.* Reconstructing ancient mitochondrial DNA links between Africa and Europe. Genome Res 22(**5**), 821 (2012). 22454235 10.1101/gr.134452.111 PMC3337428

[b31] Salas A. *et al.* A practical guide to mitochondrial DNA error prevention in clinical, forensic, and population genetics. Biochem. Biophys. Res. Commun. 335(**3**), 891 (2005). 16102729 10.1016/j.bbrc.2005.07.161

[b32] Bandelt H. -J. & Salas A. Contamination and sample mix-up can best explain some patterns of mtDNA instabilities in buccal cells and oral squamous cell carcinoma. BMC Cancer 9(**1**), 113 (2009). 19371404 10.1186/1471-2407-9-113 PMC2678148

[b33] Melchior L. *et al.* Rare mtDNA haplogroups and genetic differences in rich and poor Danish Iron-Age villages. Am J Phys Anthropol 135(**2**), 206 (2008). 18046774 10.1002/ajpa.20721

[b34] Pääbo S. Ancient DNA: extraction, characterization, molecular cloning, and enzymatic amplification. Proc Natl Acad Sci USA 86(**6**), 1939 (1989). 2928314 10.1073/pnas.86.6.1939 PMC286820

[b35] Salas A., Bandelt H. -J., Macaulay V. & Richards M. B. Phylogeographic investigations: The role of trees in forensic genetics. Forensic Sci. Int. 168(**1**), 1 (2007). 16814504 10.1016/j.forsciint.2006.05.037

[b36] Bandelt H. J., Salas A. & Lutz-Bonengel S. Artificial recombination in forensic mtDNA population databases. Int. J. Legal Med. 118(**5**), 267 (2004). 15257464 10.1007/s00414-004-0455-2

[b37] Kong Q. -P. *et al.* Distilling artificial recombinants from large sets of complete mtDNA genomes. PLoS One 3(**8**), e3016 (2008). 18714389 10.1371/journal.pone.0003016 PMC2515346

[b38] Bandelt H. -J., Salas A. & Bravi C.M. Problems in FBI mtDNA database. Science 305(**5689**), 1402 (2004). 15353782 10.1126/science.305.5689.1402b

[b39] Bandelt H. -J., Salas A., Taylor R. W. & Yao Y. -G. The exaggerated status of “novel” and “pathogenic” mtDNA sequence variants due to inadequate database searches. Hum Mutat 30(**2**), 191 (2009). 18800376 10.1002/humu.20846

[b40] Yang Z. PAML: a program package for phylogenetic analysis by maximum likelihood. Computer applications in the biosciences : CABIOS 13(**5**), 555 (1997). 9367129 10.1093/bioinformatics/13.5.555

[b41] Saillard J. *et al.* mtDNA variation among Greenland Eskimos: the edge of the Beringian expansion. Am. J. Hum. Genet. 67(**3**), 718 (2000). 10924403 10.1086/303038 PMC1287530

[b42] Slatkin M. Gene genealogies within mutant allelic classes. Genetics 143(**1**), 579 (1996). 8722806 10.1093/genetics/143.1.579 PMC1207290

[b43] Soares P. *et al.* Correcting for purifying selection: an improved human mitochondrial molecular clock. Am. J. Hum. Genet. 84(**6**), 740 (2009). 19500773 10.1016/j.ajhg.2009.05.001 PMC2694979

[b44] Drummond A. J., Rambaut A., Shapiro B. & Pybus O. G. Bayesian coalescent inference of past population dynamics from molecular sequences. Mol Biol Evol 22(**5**), 1185 (2005). 15703244 10.1093/molbev/msi103

[b45] Drummond A. J. & Rambaut A. BEAST: Bayesian evolutionary analysis by sampling trees. BMC Evol Biol 7, 214 (2007). 17996036 10.1186/1471-2148-7-214 PMC2247476

[b46] Drummond A. J., Ho S. Y., Phillips M. J. & Rambaut A. Relaxed phylogenetics and dating with confidence. PLoS Biol 4(**5**), e88 (2006). 16683862 10.1371/journal.pbio.0040088 PMC1395354

[b47] Drummond A. J., Suchard M. A., Xie D. & Rambaut A. Bayesian phylogenetics with BEAUti and the BEAST 1.7. Mol Biol Evol 29(**8**), 1969 (2012). 22367748 10.1093/molbev/mss075 PMC3408070

[b48] Fagundes N. J. *et al.* Mitochondrial population genomics supports a single pre-Clovis origin with a coastal route for the peopling of the Americas. Am. J. Hum. Genet. 82(**3**), 583 (2008). 18313026 10.1016/j.ajhg.2007.11.013 PMC2427228

[b49] Schobinger J. *et al.* El santuario incaico del Cerro Aconcagua. (Ediunc, Universidad Nacional de Cuyo, Mendoza, Argentina, 2001).

[b50] Barbieri C. *et al.* Between Andes and Amazon: the genetic profile of the Arawak-speaking Yanesha. Am J Phys Anthropol 155(**4**), 600 (2014). 25229359 10.1002/ajpa.22616

[b51] Batai K. & Williams S. R. Mitochondrial variation among the Aymara and the signatures of population expansion in the central Andes. Am J Hum Biol 26(**3**), 321 (2014). 24449040 10.1002/ajhb.22507 PMC4289594

[b52] Taboada-Echalar P. *et al.* The genetic legacy of the pre-Colonial period in contemporary Bolivians. PLoS One 8(**3**), e58980 (2013). 23527064 10.1371/journal.pone.0058980 PMC3604014

