# Supplementary material for: The complete mitogenome of a 500-year-old Inca child mummy
Source: Sci Rep. 2015 Nov 12;5:16462. doi: 10.1038/srep16462 (PMC4642457; doi:10.1038/srep16462)
Supplement: Supplementary Information [file srep16462-s1.pdf]

The complete mitogenome of a 500-year-old Inca child mummy

Alberto Gómez-Carballa, Laura Catelli, Jacobo Pardo-Seco, Federico Martinón-Torres, Carlos Vullo, Antonio Salas

Contents

Figure S1. Full C1b phylogeny. This Figure is provided in a separate file.

Figure S2. Number of mutations from the root of haplogroup C1b and their relative frequency. The red dot indicates the value for the mummy's haplotype.

Figure S3. Agarose gels of PCR amplicons of the mummy's mtDNA (+) and their corresponding negative controls (-).

Text S1. Additional information on the lab protocol for DNA extraction.

Table S1. Entire genomes belonging to haplogroup C1b. Haplogroup classification was performed according to Phylotree 16 (new haplogroups are represented in italics). Note that only coding region data are available for mitogenomes EF657584 and EF657282.

Table S2. Mitochondrial DNA haplotypes of the operators.

Figure S2.

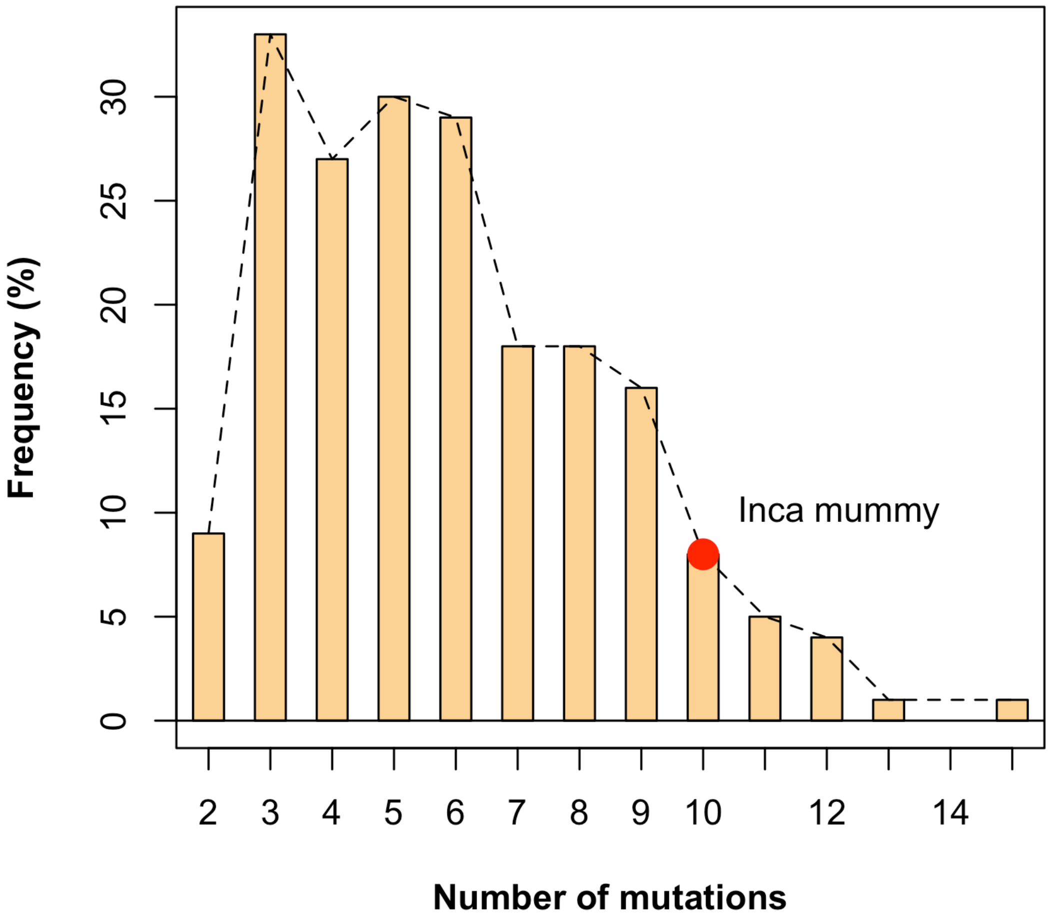

Inca mummy
Frequency (%)
0
5
10
15
20
25
30
2
3
4
5
6
7
8
9
10
12
14
Number of mutations

Figure S3.

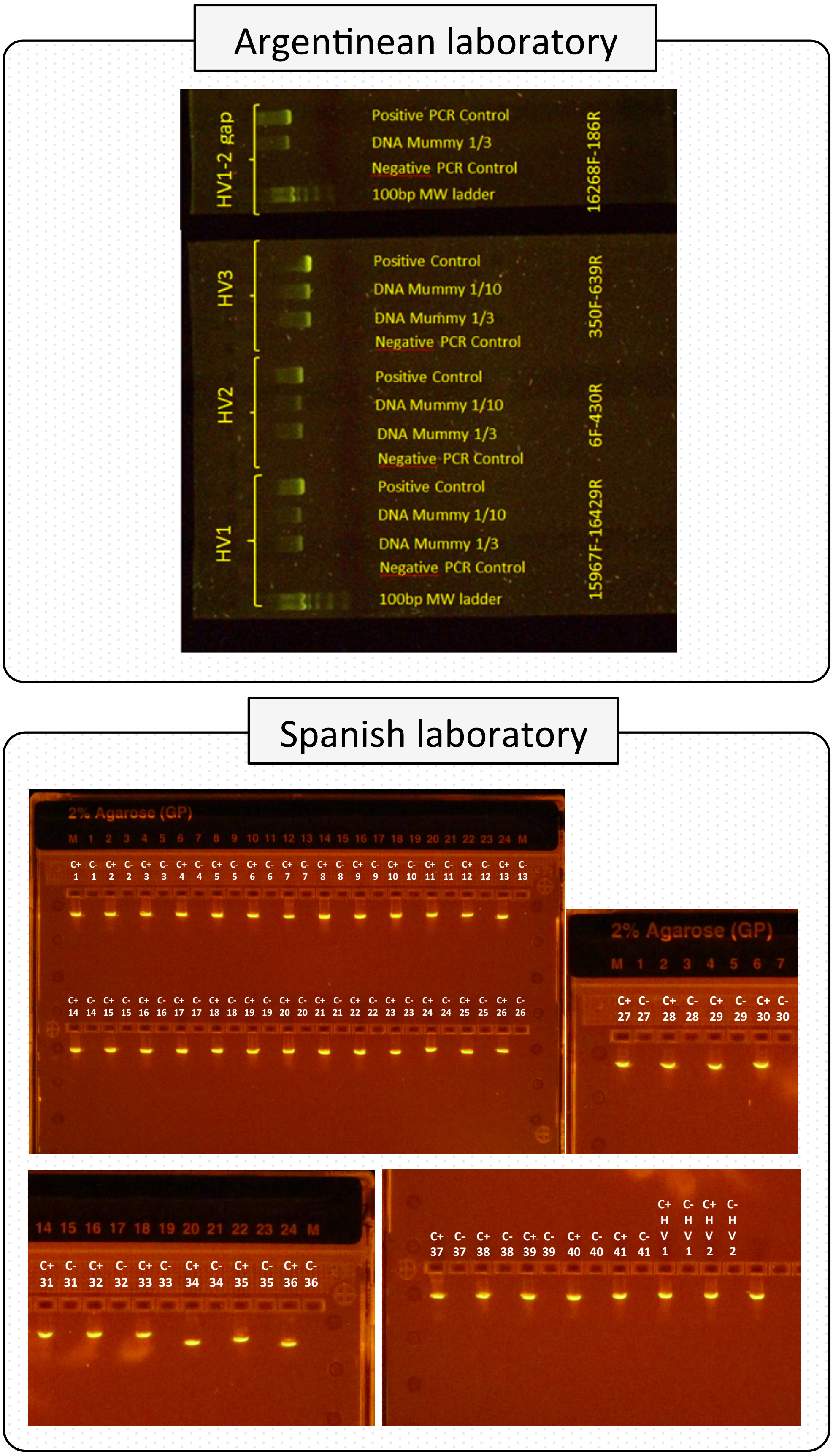

Argentinean laboratory
HV1-2 gap
Positive PCR Control
DNA Mummy 1/3
Negative PCR Control
100bp MW ladder
16268F-186R
HV3
Positive Control
DNA Mummy 1/10
DNA Mummy 1/3
Negative PCR Control
350F-639R
HV2
Positive Control
DNA Mummy 1/10
DNA Mummy 1/3
Negative PCR Control
6F-430R
HV1
Positive Control
DNA Mummy 1/10
DNA Mummy 1/3
Negative PCR Control
100bp MW ladder
15967F-16429R
Spanish laboratory
2% Agarose (GP)
2% Agarose (GP)

Text S1.

The sample was processed using a QIAMP DNA Blood Maxi kit column (Qiagen, Hilden, Germany) with substantial protocol modifications to manufacturer's recommendations. A 5ml volume of ATL buffer and 150µl of Proteinase K were added, the tube was then vortexed for 20s and later incubated inside a thermoblock for 18hs at 56ºC under agitation. An orange color was observed immediately after adding the buffer. Once the established time for incubation with digestion buffer was reached, 5ml of AL buffer and 150µl of Proteinase K were added, vortexed for 20s and then incubated for one hour at 70ºC under agitation. The whole sample was dissolved after treating it with both buffers and a strongly colored brown digest was obtained. The sample was centrifuged for 15 minutes at 2000×g in order to separate the undissolved material that may have remained in the solution. The digest was transferred to a tube containing 10ml of absolute ethanol; strongly vortexed and finally, 10ml of the digest-ethanol mixture were passed through the silica column where they were centrifuged for 3min at 1850×g in a swing-out rotor. The flow through was discarded and the remaining 10ml of the digest-ethanol mixture were again passed through the column and centrifuged. After passing the digest through the column, a strong brown color was observed in the silica. A 5ml volume of AW1 buffer was added and the mixture was centrifuged for 1min at maximum speed (3200×g) in order to remove cell debris and inhibitors. The liquid that eluted from the column after this first wash was rather colored; for this reason, two more washes were performed with the same buffer. Later, 7.5ml of AW2 buffer were added and the tube was centrifuged for 20min at maximum speed. In order to elute the DNA from the silica, 750µl of TE were added, incubated at room temperature for 5min and centrifuged for 2min at maximum speed. In order to increase the quantity of recovered DNA, a second elution round was performed but the centrifugation time was increased this time from 2 to 5min at maximum speed. The 1.5ml volume of DNA obtained was concentrated to approximately 150µl with a Vivacon 2-100MWCO ultra filtration device (Sartorius Stedim Biotech GmbH, Goettingen, Germany), and centrifuged for 4min at 2000×g to elute the greatest proportion of the DNA volume. One-minute

centrifugation cycles at 2000×g were repeated until reaching the approximate desired volume. The sample took long to pass through Vivacon (12min). Once the desired volume was reached, the ultra filtration device was turned upside down and centrifuged for 2min at 3000×g so that the DNA was obtained in the smaller cap. Since the extract showed a light straw color, additional purifications were performed using Qiaquick columns (Qiagen) to remove possible PCR inhibitors such as Maillard products.

Table S1.

Reference	Genbank acc. nº	Ethnic group	Geographic origin	Haplogroup	Haplotypes
[5]	EU095225	Quechua	-	C1b	73 249d 263 290-291d 309+CC 315+C 489 493 523-524d 750 1438 2706 3552A 4014 4491 4715 4769 7028 7196A 8584 8701 8860 9540 9545 10398 10400 10873 11719 11914 12705 13263 13656 14318 14766 14783 15043 15301 15326 15487T 16199 16223 16254 16298 16325 16327 16519
[5]	EU095226	Quechua	-	C1b	73 249d 263 290-291d 309+CC 315+C 489 493 523-524d 750 1438 2706 3552A 4715 4769 6456 7028 7196A 8584 8701 8860 9540 9545 10398 10400 10873 11719 11914 12705 13263 13545 14318 14766 14783 15043 15301 15326 15487T 16223 16298 16318C 16325 16327 16519
[5]	EU095227	Arara/Arara do Laranjal	Brazil?	C1b	73 249d 263 290-291d 309+CC 315+C 489 493 523-524d 750 960+XC 1438 2706 3552A 3764 4715 4769 4924 7028 7196A 8078 8584 8701 8860 9540 9545 10398 10400 10873 11719 11914 12705 13263 14318 14766 14783 15043 15301 15326 15487T 16223 16292 16298 16325 16327 16362
[5]	EU095228	Poturujara	Brazil?	C1b	73 195 249d 263 290-291d 309+C 315+C 489 493 523-524d 750 1438 2706 3552A 4715 4769 5238 7028 7196A 8584 8701 8860 9540 9545 10398 10400 10873 11719 11914 12705 13263 14318 14766 14783 14788 15043 15301 15326 15487T 16298 16325 16327 16519
[6]	EU095549	Wayuu	Guajira peninsula	C1b	73 249d 263 290-291d 309+CC 315+C 485 489 493 523-524d 750 1438 2706 3552A 3768 4715 4769 5600 6872 7028 7196A 8584 8701 8860 9540 9545 10398 10400 10873 11719 11914 12705 13263 13734 14318 14766 14783 15043 15301 15326 15465 15487T 16223 16298 16325 16327
taken from 1000Genomes	HG01571	-	Peru (Lima)	C1b	73 204 249d 263 290-291d 309+CC 315+C 489 493 523-524d 750 1438 2706 3552A 4715 4769 5231 7028 7196A 8584 8701 8860 9540 9545 9827 10398 10400 10873 11719 11914 12705 12850 13263 14318 14766 14783 15043 15301 15315 15326 15487T 16172 16223 16298 16319 16325 16519
taken from 1000Genomes	HG01932	-	Peru (Lima)	C1b	73 235 249d 263 290-291d 315+C 489 493 523-524d 750 1438 1811 2371 2706 3552A 4715 4769 6662 7028 7196A 8584 8701 8860 8943 9540 9545 10398 10400 10873 11311 11719 11914 12705 13263 13711 14318 14766 14783 15043 15301 15326 15487T 15808 16086 16176 16192 16223 16298 16325 16327
taken from 1000Genomes	HG01948	-	Peru (Lima)	C1b	73 249d 263 290-291d 309+C 315+C 489 493 523-524d 750 1438 2706 3552A 4715 4769 7013 7028 7196A 8584 8701 8860 9540 9545 10310 10398 10400 10873 11719 11914 12705 13263 14181 14318 14488 14766 14783 15043 15301 15326 15487T 16223 16298 16325 16327
taken from 1000Genomes	HG01961	-	Peru (Lima)	C1b	73 249d 263 290-291d 309+C 315+C 489 493 523-524d 750 1438 2706 3552A 4715 4769 5021 7028 7196A 8074 8479 8584 8701 8860 9540 9545 9947 10398 10400 10873 11719 11914 12705 13263 14318 14766 14783 15043 15301 15326 15487T 16223 16298 16325 16327
taken from 1000Genomes	HG01965	-	Peru (Lima)	C1b	73 249d 290-291d 315+C 489 493 523-524d 521T 750 1438 2706 3552A 4715 4769 7028 7196A 8404 8584 8701 8860 9337 9540 9545 10398 10400 10873 11719 11914 12705 13263 14318 14494 14766 14783 15043 15301 15326 15487T 15844 16180 16223 16298 16325 16327
taken from 1000Genomes	HG01982	-	Peru (Lima)	C1b	73 215 235 249d 263 290-291d 309+C 315+C 489 493 523-524d 750 1438 2380 2706 3552A 4715 4769 5582 7028 7193 7196A 8574G 8584 8701 8860 9540 9545 9755 10306C 10398 10400 10873 11719 11914 12705 13263 13711 14318 14766 14783 15043 15301 15326 15487T 16183d 16183C 16189 16223 16298 16325 16327 16428 16430
taken from 1000Genomes	HG02265	-	Peru (Lima)	C1b	73 195 249d 263 290-291d 315+C 489 493 523-524d 750 1438 2706 3552A 4715 4769 7028 7196A 7244 8584 8604 8701 8860 9540 9545 10398 10400 10873 11719 11914 12705 13263 13980C 14318 14766 14783 15043 15301 15326 15487T 16223 16298 16325 16327
taken from 1000Genomes	HG02278	-	Peru (Lima)	C1b	73 195 249d 263 290-291d 309+CC 315+C 489 493 523-524d 750 1407 1438 2272 2706 3552A 4715 4769 6794 7028 7196A 8584 8701 8860 9084 9540 9545 9923 10398 10400 10873 11719 11914 12020 12705 13263 13579 14318 14766 14783 15043 15301 15326 15487T 16129 16223 16298 16325 16327 16357 16380
[9]	HQ012200	Mexica American	Central America	C1b	72 73 194 249d 263 290-291d 315+C 489 493 523-524d 750 1438 2706 3552A 4715 4769 6480 7028 7196A 8584 8701 8860 9540 9545 10398 10400 10873 11719 11914 12705 13263 14053 14318 14766 14783 15043 15301 15326 15487T 16187 16223 16298 16325 16327
[9]	HQ012209	Mexica American	Central America	C1b	73 152 199 207 249d 263 290-291d 309+C 315+C 340 489 493 523-524d 750 1438 2706 2757 3552A 4715 4769 7028 7196A 8508 8584 8701 8860 9500 9540 9545 10398 10400 10873 11719 11914 12705 13263 13830 14318 14766 14783 15043 15301 15326 15487T 16092 16223 16298 16325 16327
[9]	HQ012211	Mexica American	Central America	C1b	9 73 125G 199 249d 263 290-291d 315+C 489 493 523-524d 750 1438 2706 3531 3552A 4715 4769 5057 7028 7196A 8584 8701 8860 9540 9545 10398 10400 10873 11719 11914 12705 13263 14318 14766 14783 15043 15301 15326 15487T 16176 16223 16298 16325 16327
[10]	JQ702185	-	-	C1b	73 143 195 249d 263 290-291d 315+C 489 493 523-524d 750 1438 2706 3552A 4715 4769 6253 6635 7028 7196A 8584 8701 8860 9540 9545 10370 10398 10400 10873 11084 11719 11914 12612 12705 13263 14318 14766 14783 15043 15301 15326 15487T 16086 16223 16291 16325 16327
[10]	JQ702914	-	-	C1b	73 249d 263 290-291d 489 493 523-524d 750 1438 2706 3552A 4715 4769 7028 7196A 8584 8701 8860 9540 9545 9686 10031 10398 10400 10873 11719 11914 12705Y 12879Y 12976 13263 14318 14766 14783 15043 15301 15326 15487T 16223 16294 16298 16325 16327
[10]	JQ705665	-	-	C1b	73 249d 263 290-291d 489 493 523-524d 750 1438 2706 3552A 4715 4769 7028 7196A 8584 8701 8860 9540 9545 9686 10031 10398 10400 10873 11719 11914 12705 12879 12976 13263 14318 14766 14783 15043 15301 15326 15487T 16223 16294 16298 16325 16327
Unpublised data	JX669186	-	Peru (Huancayo)	C1b	73 249d 263 290-291d 309+C 315+C 489 493 523-524d 750 1438 2706 3552A 4715 4769 7028 7196A 8584 8701 8860 9540 9545 10398 10400 10873 11150 11719 11914 12705 13263 14318 14766 14783 15043 15301 15326 15487T 16223 16298 16325 16327 16390 16519
Unpublised data	JX669188	-	Peru (Huancayo)	C1b	73 249d 263 290-291d 309+C 315+C 489 493 523-524d 750 1438 2706 3552A 4715 4769 7028 7196A 8584 8701 8860 9540 9545 10398 10400 10873 11150 11719 11914 12705 13263 14318 14766 14783 15043 15301 15326 15487T 16223 16298 16325 16327 16390 16519
Unpublised data	JX669189	-	Peru (Huancayo)	C1b	60 73 249d 263 290-291d 315+C 489 493 523-524d 750 1438 2706 3552A 4117 4715 4769 7028 7196A 8584 8701 8860 9540 9545 10398 10400 10873 11719 11914 12705 13263 14318 14766 14783 15043 15301 15326 15487T 15574 16223 16298 16325 16327 16356
Unpublised data	JX669192	-	Peru (Huancayo)	C1b	73 185 249d 263 290-291d 309+C 315+C 489 493 523-524d 750 1438 2706 3552A 4715 4769 6253 6480 6494 7028 7196A 8131 8584 8701 8860 9540 9545 10398 10400 10873 11296 11447 11719 11914 12705 13263 14318 14766 14783 14833 15043 15301 15326 15487T 16114 16181 16189 16223 16298 16325 16327 16344
Unpublised data	JX669195	-	Peru (Huancayo)	C1b	73 204 249d 263 290-291d 310 311+T 489 493 523-524d 750 1438 1462 2706 3552A 3866 4715 4769 7028 7196A 8584 8701 8860 9540 9545 10398 10400 10873 11719 11914 12705 12975 13263 13860 14318 14602 14766 14783 15043 15301 15326 15487T 16037 16223 16298 16325 16327
Unpublised data	JX669212	-	Peru (Lima)	C1b	73 214 249d 263 290-291d 309+C 315+C 489 493 523-524d 750 1438 2370 2706 3552A 4715 4769 5201A 5824 7028 7196A 8584 8701 8860 9540 9545 10398 10400 10873 11719 11914 12705 13263 14318 14766 14783 15043 15301 15326 15487T 16145 16192 16223 16298 16325 16327 16519

Unpublised data	JX669214	-	Peru (Lima)	C1b	73 146 249d 263 290-291d 309+C 315+C 489 493 523-524d 750 1438 2706 3552A 4454 4715 4769 5628 6267 7028 7196A 8584 8634 8701 8860 8901 9540 9545 10398 10400 10873 11719 11914 12705 13263 14318 14766 14783 15043 15301 15326 15487T 16092 16223 16256 16298 16325 16327
Unpublised data	JX669246	-	Peru (Lima)	C1b	73 249d 263 290-291d 315+C 489 493 523-524d 750 1040 1438 2706 3552A 4715 4769 6267 7028 7196A 8584 8701 8860 9540 9545 10398 10400 10873 11410 11719 11914 12302 12705 13263 14318 14766 14783 15043 15301 15326 15487T 15758 16223 16298 16325 16327
Unpublised data	JX669247	-	Peru (Lima)	C1b	73 249d 263 290-291d 309+C 315+C 489 493 523-524d 750 1438 2706 3552A 4715 4769 7028 7196A 8584 8701 8860 9084 9540 9545 10398 10400 10873 10993 11431A 11719 11914 12705 13263 14318 14384 14766 14783 15043 15301 15326 15487T 15758 16129 16223 16298 16325 16327 16519
Unpublised data	JX669254	-	Peru (Lima)	C1b	73 249d 263 290-291d 309+C 315+C 489 493 523-524d 573+XC 750 1438 2706 3552A 4715 4769 7028 7196A 8584 8701 8860 9540 9545 10398 10400 10873 11719 11914 12705 13263 13482T 13695A 14318 14766 14783 15043 15301 15326 15487T 16223 16298 16325 16327 16390 16519
Unpublised data	JX669264	-	Peru (Lima)	C1b	73 249d 263 290-291d 309+C 315+C 489 493 523-524d 750 1438 2706 3552A 3882 4715 4769 7028 7196A 8584 8701 8860 9540 9545 10398 10400 10873 11719 11914 12705 13263 14318 14659 14766 14783 15043 15301 15326 15487T 16223 16298 16325 16327 16519
Unpublised data	JX669268	-	Peru (Lima)	C1b	73 249d 263 290-291d 309+CC 315+C 489 493 523-524d 750 1438 2706 3552A 4715 4769 5580 7028 7196A 7354 8566 8584 8701 8860 9210 9540 9545 10398 10400 10601 10873 11719 11914 12705 12717 13263 14318 14766 14783 15043 15301 15326 15487T 15970 16223 16298 16325 16327 16519
Unpublised data	JX669275	-	Peru (Lima)	C1b	73 249d 263 290-291d 309+C 315+C 489 493 523-524d 750 1391 1438 2626G 2706 3552A 4715 4769 4994 5460 6959 7028 7196A 8516 8584 8701 8860 9540 9545 10398 10400 10873 11719 11914 12705 13263 14318 14766 14783 15043 15301 15326 15487T 15968 16071 16093 16140 16223 16298 16325 16327 16445
Unpublised data	JX669278	-	Peru (Lima)	C1b	73 249d 263 290-291d 315+C 489 493 523-524d 723C 750 1438 2706 3552A 4715 4769 7028 7196A 8584 8701 8860 9540 9545 10398 10400 10873 11719 11914 12705 13263 14318 14706 14766 14783 15043 15301 15326 15487T 16147A 16223 16298 16325 16327
Unpublised data	JX669280	-	Peru (Lima)	C1b	73 125 127 235 249d 263 290-291d 315+C 489 493 523-524d 750 1438 2706 3552A 4715 4769 7028 7196A 8584 8701 8860 9540 9545 9755 10398 10400 10873 11026 11719 11914 12705 13135 13263 13711 14318 14766 14783 15043 15301 15326 15454 15487T 15970 16223 16325 16327
Unpublised data	JX669294	-	Peru (Lima)	C1b	73 249d 263 290-291d 309+C 315+C 489 493 523-524d 750 1040 1438 2706 3552A 4715 4769 6267 7028 7196A 8701 8860 9316 9540 9545 10398 10400 10420 10873 11410 11719 11914 12302 12705 13263 14318 14766 14783 15043 15301 15326 15487T 15758 16223 16298 16325 16327
Unpublised data	JX669306	-	Peru (Lima)	C1b	73 249d 263 290-291d 309+C 315+C 489 493 523-524d 750 1040 1438 2706 3552A 4715 4769 6267 7028 7196A 8584 8701 8860 9316 9540 9545 10398 10400 10420 10873 11410 11719 11914 12302 12705 13263 14318 14766 14783 15043 15301 15326 15487T 15758 16223 16294Y 16298 16325 16327
Unpublised data	JX669313	-	Peru (Lima)	C1b	73 249d 263 290-291d 315+C 489 493 523-524d 750 1438 2706 3552A 4715 4769 7028 7196A 8584 8701 8860 9540 9545 10398 10400 10873 11719 11914 12705 13263 14122 14318 14766 14783 15043 15301 15326 15346 15355 15487T 16051 16223 16297 16298 16325 16327
Unpublised data	JX669333	-	Peru (Lima)	C1b	73 235 249d 263 290-291d 315+C 489 493 523-524d 750 1438 2706 3552A 4715 4769 6253 6569 7028 7196A 8584 8701 8860 9540 9545 10398 10400 10873 11719 11914 12705 13263 14122 14318 14766 14783 15043 15301 15326 15487T 16051 16192 16223 16298 16325 16327 16519
Unpublised data	JX669356	-	Peru (Lima)	C1b	73 143T 249d 263 290-291d 309+CC 315+C 489 493 523-524d 750 980 1438 1808 2706 3552A 4715 4769 5147 7028 7196A 8584 8701 8860 9540 9545 10398 10400 10873 11389 11719 11914 12705 13263 14318 14766 14783 15043 15301 15326 15487T 16145 16223 16298 16325 16327 16471
Unpublised data	JX669367	-	Peru (Lima)	C1b	73 185 249d 263 290-291d 309+CC 315+C 489 493 523-524d 750 1438 2706 3552A 4715 4769 6253 6480 7028 7196A 8584 8701 8860 9329 9540 9545 10398 10400 10873 11719 11914 12705 12717 13263 14302 14318 14374 14766 14783 14833 15043 15301 15326 15487T 16126 16183C 16189 16223 16298 16325 16327 16344 16519
Unpublised data	JX669380	-	Peru (Lima)	C1b	73 185 249d 263 290-291d 309+CC 315+C 489 493 523-524d 750 1438 2706 3552A 4715 4769 6253 6480 7028 7196A 8584 8701 8860 9540 9545 10398 10400 10873 11719 11914 12705 12717 13263 14302 14318 14374 14766 14783 14833 15043 15301 15326 15487T 16126 16183C 16189 16223 16298 16325 16327 16344 16519
Unpublised data	JX669382	-	Peru (Lima)	C1b	73 188 249d 263 290-291d 315+C 489 493 750 1438 2706 3357 3552A 4715 4769 5417 7028 7196A 8584 8701 8860 9540 10398 10400 10798 10873 11243 11719 11914 12705 13263 14318 14766 14783 15043 15301 15326 15487T 16209 16223 16234 16298 16325 16327
Unpublised data	JX669416	-	Peru (Ucayali)	C1b	73 152 249d 263 290-291d 309+C 315+C 489 493 523-524d 750 1438 1692C 2706 3552A 4715 4769 7028 7076 7196A 8584 8701 8860 9329 9540 9545 10398 10400 10873 11719 11914 12705 13263 13368 14318 14766 14783 15043 15301 15326 15487T 16146 16223 16298 16325 16327
Famlily tree	KC150027	Brazilian	Brazil (Bahia)	C1b	71 73 249d 263 290-291d 309+CC 315+C 489 493 523-524d 750 1438 2393 2706 3197 3394 3552A 4715 4769 7028 7196A 8584 8701 8860 9540 9545 10398 10400 10873 11719 11914 12705 13263 13766A 14318 14766 14783 15043 15301 15326 15487T 16126 16223 16270 16298 16325 16327
[13]	KJ923818	Guarani	Brazil	C1b	45 64 73 249d 263 290-291d 309N 310 489 493 523-524d 750 1131N 1147N 1151N 1173N 1185N 1197N 1211A 1227N 1230N 1231N 1249N 1257N 1263N 1283N 1284N 1285d 1292N 1295N 1303N 1310N 1312N 1315N 1320N 1438 2056N 2071N 2706 3552A 4715 4736 4769 5054C 6249 7028 7196A 8134 8474A 8584 8701 8860 9540 9545 10398 10400 10873 11719 11914 12705 13263 14118 14318 14766 14783 15043 15301 15326 15487T 15670 16223 16325 16327
[13]	KJ923823	Inga	Colombia	C1b	73 249d 263 290-291d 30+C 315+C 489 493 523-524d 750 1438 2706 3552A 4529 4769 7028 7184 7196A 8584 8701 8860 9540 9545 10398 10400 10873 11719 11914 12705 13263 14318 14766 14783 15043 15301 15326 15487T 16223 16298 16325 16327 16519
[13]	KJ923844	Wayuu	Guajira peninsula	C1b	73 249d 263 290-291d 309+C 315+C 485 489 493 523-524d 750 1438 2706 3552A 3768 4715 4769 5600 6872 7028 7196A 8584 8701 8860 9540 9545 10398 10400 10873 11719 11914 12705 13263 13734 14318 14766 14783 15043 15301 15326 15465 15487T 16223 16298 16325 16327
Family tree	KP851974	-	Ecuador	C1b	73 249d 263 290-291d 315+C 489 493 523-524d 750 1438 1709 2706 3552A 4715 4769 7028 7196A 8584 8701 8860 9540 9545 10364 10398 10400 10873 11719 11914 12705 13263 14318 14766 14783 15043 15301 15326 15487T 15924 16223 16298 16325 16327 16519
taken from 1000Genomes	NA19726	Mexica American	USA (California)	C1b	73 249d 263 290-291d 309+C 315+C 489 493 523-524d 750 1438 2345 2706 3552A 4715 4769 7028 7196A 7211 8584 8701 8860 9540 9545 10398 10400 10873 11152 11560 11719 11914 12705 13263 14318 14766 14783 15043 15301 15326 15487T 16223 16298 16325 16327
This study		INCA	Argentina (Cerro Aconcagua; Andes)	C1b	56T 57 60+T 64 73 249d 263 290-291d 309+C 315+C 455+T 489 493 523-524d 662 750 1438 2563 2706 3552A 4715 4769 5135 7028 7196A 8584 8701 8725 8860 9540 9545 10398 10400 10873 11719 11914 12705 13263 14318 14766 14783 15043 15301 15326 15487T 16124 16223 16298 16325 16327 16519
Unpublised data	JX669185	-	Peru (Huancayo)	C1b+16311	73 249d 263 290-291d 309+C 315+C 489 493 523-524d 750 1438 2280 2706 3552A 3816 4715 4769 7028 7196A 8584 8701 8860 9540 9545 10398 10400 10873 11719 11914 12705 13263 14318 14325Y 14766 14783 15043 15301 15326 15487T 16223 16298 16311 16325 16327

Unpublised data	JX669386	-	Peru (Lima)	C1b+16311	73 235 249d 263 290-291d 315+C 489 493 512 523-524d 750 961 965+XC 1438 2706 3010 3552A 4715 4769 4928 7028 7196A 8584 8701 8860 9254 9540 9545 9755 10398 10400 10873 11026 11719 11914 12173 12705 13263 13711 14318 14766 14783 15043 15301 15326 15355 15487T 16223 16274 16294 16298 16311 16325 16327
Unpublised data	JX669390	-	Peru (Lima)	C1b+16311	73 146 150 249d 263 290-291d 309+C 315+C 489 493 523-524d 750 1438 2706 3552A 4715 4769 7028 7196A 7521 8584 8701 8860 9540 9545 10398 10400 10873 11719 11914 12705 13263 13943 14318 14766 14783 15043 15301 15326 15487T 16223 16298 16311 16325 16327
taken from 1000Genomes	NA20299	African Ancestry	USA (South west)	C1b+16311	73 249d 259 263 290-291d 315+C 489 493 523-524d 573+CC 750 1438 2706 3552A 4769 5981 7028 7196A 8584 8701 8860 9389 9540 9545 10094 10398 10400 10873 11719 11914 12705 13263 14318 14766 14783 15043 15301 15326 15487T 15670 16126 16223 16298 16311 16325 16327 16519
[9]	HQ012198	Mexica American	Central America	C1b10	73 146 249d 263 290-291d 309+C 315+C 385 489 493 523-524d 750 1438 2706 3552A 4715 4769 6284 7028 7196A 8584 8701 8860 9540 9545 10398 10400 10873 11719 11914 12705 13263 14318 14766 14783 15043 15301 15326 15487T 16129 16172 16223 16298 16311 16325 16327
[9]	HQ012188	Mexica American	Central America	*C1b10a*	73 146 152 249d 263 290-291d 309+C 315+C 385 489 493 523-524d 750 1438 2706 3552A 4715 4769 6284 7028 7196A 8584 8701 8860 9540 9545 10398 10400 10873 11719 11914 12705 13263 14318 14482 14766 14783 15043 15301 15326 15487T 15622 16129 16172 16223 16298 16311 16325 16327 16519
[9]	HQ012206	Mexica American	Central America	*C1b10a*	73 146 152 249d 263 290-291d 309+C 315+C 385 489 493 523-524d 750 857 1438 2706 3552A 4715 4769 6284 7028 7196A 8584 8701 8860 9540 9545 10398 10400 10873 11719 11914 12705 13263 14318 14482 14766 14783 15043 15301 15326 15487T 15622 16129 16172 16223 16298 16311 16325 16327 16519
[9]	HQ012214	Mexica American	Central America	*C1b10a*	73 146 152 249d 263 290-291d 310 316C 385 489 493 523-524d 750 1438 2706 3552A 4715 4769 6284 7028 7196A 8584 8701 8860 9099 9540 9545 10398 10400 10873 11719 11914 12705 13263 14318 14482 14766 14783 15043 15301 15326 15487T 15622 16129 16172 16183C 16189 16223 16298 16311 16325 16327 16519
[9]	HQ012217	Mexica American	Central America	*C1b10a*	73 146 152 249d 263 290-291d 315+C 385 489 493 523-524d 750 1438 2706 3552A 4715 4769 5414 6284 7028 7196A 8584 8701 8860 9540 9545 10398 10400 10873 11719 11914 12705 13263 14318 14482 14766 14783 15043 15301 15326 15487T 15622 16129 16172 16223 16298 16311 16325 16327 16519
[10]	JQ705826	-	-	*C1b10b*	73 249d 290-291d 309+C 315+C 385 489 493 523-524d 750 1438 2706 3552A 4715 4769 6284 7028 7196A 8584 8701 8860 9540 9545 10184 10398 10400 10873 11719 11914 12705 13263 14318 14766 14783 15043 15301 15326 15487T 15772 16129 16172 16181 16189 16298 16311 16325 16327
[10]	JQ705920	-	-	*C1b10b*	73 249d 290-291d 309+C 315+C 385 489 493 523-524d 750 1438 2706 3552A 4715 4769 6284 7028 7196A 8584 8701 8860 9540 9545 10184 10398 10400 10873 11719 11914 12705 13263 14318 14766 14783 15043 15301 15326 15487T 16129 16172 16181 16189 16298 16311 16325 16327
[10]	JQ701891	-	-	C1b11	73 249d 263 290-291d 309+C 315+C 489 493 523-524d 750 1438 1517 2706 3397 3552A 4370 4715 4769 5899+XC 7028 7196A 8334 8584 8701 8860 9540 9545 10398 10400 10873 11719 11914 12705 13263 14318 14766 14783 15043 15301 15326 15487T 16223 16295 16298 16325 16327 16433 16519
taken from 1000Genomes	NA19658	Mexica American	USA (California)	C1b11	73 194 249d 263 290-291d 309+C 315+C 489 493 523-524d 750 1438 2706 3084 3552A 4715 4769 7028 7196A 8584 8701 8860 9540 9545 10398 10400 10873 11719 11914 12705 13263 14318 14766 14783 15043 15301 15326 15487T 16223 16295 16298 16325 16327
[10]	JQ705451	-	-	*C1b11a*	73 194 249d 263 290-291d 309+C 315+C 489 493 523-524d 750 1438 2706 3552A 4715 4769 4925 7028 7196A 8584 8701 8860 9540 9545 10398 10400 10514 10873 11719 11914 12705 13263 14318 14766 14783 15043 15301 15326 15487T 16223 16295 16298 16325 16327
[8]	EU597545	PIMA	Mexico	*C1b11a1*	61 62C 63G 73 194 249d 263 290-291d 309+C 315+C 489 493 523-524d 750 1438 2706 3552A 4715 4769 7028 7196A 7757 8584 8701 8860 9540 9545 10398 10400 10514 10873 11719 11914 12705 13263 14318 14766 14783 15043 15301 15326 15487T 15924 16223 16295 16298 16325 16327
[8]	EU597557	PIMA	Mexico	*C1b11a1*	61 62C 63G 73 194 249d 263 290-291d 309+C 315+C 489 493 523-524d 750 1438 2706 3552A 4715 4769 7028 7196A 7757 8584 8701 8860 9540 9545 10398 10400 10514 10873 11719 11914 12705 13263 14318 14766 14783 15043 15301 15326 15487T 15924 16223 16295 16298 16325 16327
[10]	JQ704210	-	-	*C1b11a1*	63d 73 194 249d 263 290-291d 309+CC 315+C 489 493 523-524d 750 1438 2706 3552A 4715 4769 7028 7196A 8584 8701 8860 9540 9545 9554 10398 10400 10514 10873 11719 11914 12705 13263 14318 14766 14783 15043 15301 15326 15487T 15924 16223 16295 16298 16325 16327
[10]	JQ702079	-	-	*C1b11b*	72 73 194 249d 263 290-291d 309+C 315+C 489 493 523-524d 750 1438 2706 3552A 4715 4769 6086 7028 7196A 8149 8584 8701 8848 8860 9540 9545 10398 10400 10873 11719 11914 12172 12705 13263 14318 14766 14783 15043 15301 15326 15487T 16223 16295 16298 16311 16325 16327
[9]	HQ012203	Mexica American	Central America	*C1b11b1*	72 73 194 249d 263 290-291d 309+C 315+C 489 493 523-524d 750 1438 2706 3552A 4715 4769 6086 7028 7196A 8149 8584 8701 8848 8860 9540 9545 10398 10400 10873 11719 11914 12172 12705 13263 14318 14766 14783 15043 15301 15326 15487T 16223 16249 16295 16298 16311 16325 16327
[10]	JQ704884	-	-	*C1b11b1*	72 73 194 249d 263 290-291d 309+C 315+C 489 493 523-524d 750 1438 2706 3552A 4715 4769 6086 7028 7196A 8149 8512 8584 8701 8848 8860 9540 9545 10398 10400 10873 11719 11914 12172 12705 13263 14318 14766 14783 15043 15301 15326 15487T 16223 16249 16295 16298 16311 16325 16327
[9]	HQ012197	Mexica American	Central America	C1b12	73 185 249d 263 290-291d 315+C 489 493 523-524d 750 1438 2706 3405 3552A 4715 4769 7028 7196A 8584 8701 8860 9456 9540 9545 10398 10400 10873 11025 11719 11914 12705 13263 14318 14766 14783 15043 15301 15326 15487T 16223 16298 16325 16327
taken from 1000Genomes	NA19657	Mexica American	USA (California)	C1b12	73 249d 263 290-291d 315+C 489 493 523-524d 750 1438 2706 3552A 4715 4769 7028 7196A 8584 8701 8860 9540 9545 10398 10400 10873 11025 11719 11914 12705 13263 14318 14766 14783 15043 15301 15326 15487T 16223 16234 16298 16325 16327 16509
taken from 1000Genomes	NA19786	Mexica American	USA (California)	C1b12	73 249d 263 290-291d 315+C 489 493 523-524d 750 1438 2706 3552A 3946 4715 4769 7028 7196A 8584 8701 8860 9540 9545 10398 10400 10873 11025 11719 11914 12705 13263 14318 14766 14783 15043 15301 15326 15487T 16223 16298 16325 16327 16509
[9]	HQ012189	Mexica American	Central America	*C1b12a*	73 249d 263 290-291d 316d 489 493 523-524d 750 1438 2706 3552A 4715 4769 7028 7196A 8584 8701 8860 9540 9545 10398 10400 10873 11025 11419 11719 11914 12406 12705 13263 14318 14766 14783 15043 15301 15326 15487T 16223 16298 16325 16327
[10]	JQ705153	-	-	*C1b12a*	73 249d 263 290-291d 315+C 489 493 523-524d 750 1438 2706 3552A 4715 4769 7028 7196A 8584 8701 8860 9540 9545 10398 10400 10873 11025 11419 11719 11914 12705 13263 14318 14766 14783 15043 15301 15326 15487T 16223 16298 16325 16327
[11]	JX413055	Kolla	Argentina (Salta)	C1b13	73 105-110d 188 249d 258 263 290-291d 315+C 489 493 523-524d 750 1438 2706 3552A 4715 4769 7028 7091 7196A 8584 8701 8860 9540 9545 10398 10400 10873 11719 11914 12705 13263 14318 14766 14783 15043 15301 15326 15487T 16223 16298 16325 16327
[11]	JX413056	Rural	Chile (Carelmapu, Chiloe Island)	C1b13	73 249d 258 263 290-291d 309+C 315+C 489 493 523-524d 750 1438 2706 3552A 4715 4769 7028 7091 7196A 8584 8701 8860 9540 9545 10398 10400 10873 11270 11719 11914 12705 13263 14318 14766 14783 15043 15301 15326 15487T 16223 16298 16311 16325 16327 16343 16519

[11]	JX413039	Urban	Chile	C1b13a	73 249d 258 263 290-291d 309+C 315+C 489 493 523-524d 750 1193 1438 2706 3552A 4715 4769 7028 7091 7196A 8584 8701 8860 9540 9545 10398 10400 10873 11719 11914 12705 13263 14318 14766 14783 15043 15301 15326 15482 15487T 16129 16207 16223 16298 16325 16327
[11]	JX413040	Urban	Chile (Los Andes, Aconcagua)	C1b13a	73 249d 258 263 290-291d 309+C 315+C 489 493 523-524d 750 1193 1438 2706 3552A 4715 4769 7028 7091 7196A 8584 8701 8860 9540 9545 10398 10400 10873 11719 11914 12705 13263 14318 14766 14783 15043 15301 15326 15482 15487T 16129 16207 16223 16298 16325 16327
[11]	JX413036	Huilliche	Chile (San Juan de la Costa)	C1b13a1	73 214 249d 258 263 290-291d 309+C 315+C 489 493 523-524d 750 1193 1438 2706 3552A 4715 4769 7028 7091 7196A 8584 8701 8860 9540 9545 10398 10400 10873 11084 11719 11914 12411 12705 13263 14318 14766 14783 15043 15301 15326 15487T 15781 15884 16223 16298 16325 16327 16471
[11]	JX413037	Urban	Chile (Los Andes, Aconcagua)	C1b13a1	73 249d 258 263 290-291d 309+C 315+C 489 493 523-524d 750 1193 1438 2706 3552A 4715 4769 5985 7028 7091 7196A 7897 8537 8584 8701 8825 8860 9540 9545 10238 10398 10400 10873 10927 11084 11719 11914 12411 12705 13263 14318 14766 14783 15043 15301 15326 15487T 16223 16298G 16325 16327 16448
[11]	JX413038	Urban	Chile (San Felipe, Aconcagua)	C1b13a1	73 249d 258 263 290-291d 309+C 315+C 489 493 523-524d 750 1193 1438 2706 3552A 4375 4715 4769 7028 7091 7196A 8584 8701 8860 9540 9545 10398 10400 10873 11084 11719 11914 12411 12705 13263 14318 14587 14766 14783 15043 15301 15326 15487T 16223 16263 16298 16325 16327 16362
[11]	JX413041	Huilliche	Chile (San Juan de la Costa)	C1b13b	73 150 249d 258 263 290-291d 315+C 489 493 523-524d 750 1438 1842 2706 3552A 4715 4769 5414 6248 7028 7091 7196A 7621 8584 8701 8860 9540 9545 10101 10398 10400 10873 11719 11914 12705 13263 14318 14766 14783 15043 15262 15301 15326 15487T 16223 16298 16311 16325 16327
[11]	JX413042	Rural	Chile (Quetalmahue, Chiloe Island)	C1b13b	73 249d 258 263 290-291d 315+C 489 493 523-524d 750 1438 1842 2706 3552A 4715 4769 7028 7091 7196A 8584 8701 8860 9540 9545 10101 10398 10400 10873 11719 11914 12705 13263 14318 14766 14783 15043 15301 15326 15487T 16219 16223 16298 16301 16325 16327
[11]	JX413043	Born in Spain	Chile (Talagante)	C1b13b	73 249d 258 263 290-291d 315+C 489 493 523-524d 750 1438 1842 2706 3552A 4452 4715 4769 5894 7028 7091 7196A 8584 8701 8860 9540 9545 10101 10398 10400 10873 11719 11914 12705 13263 14318 14766 14783 15043 15301 15326 15487T 16137 16223 16298 16325 16327
[11]	JX413046	Chilean	Chile	C1b13c1	73 249d 258 263 290-291d 309+C 315+C 489 493 750 1438 2706 3552A 4715 4769 7028 7091 7196A 8584 8701 8860 9540 9545 10398 10400 10873 11719 11914 11963 12705 13263 13392 14318 14766 14783 15043 15244 15301 15326 15487T 16223 16298 16325 16327
[11]	JX413047	Chilean	Chile	C1b13c1	73 249d 258 263 290-291d 309+C 315+C 489 493 750 1438 2706 3552A 4715 4769 7028 7091 7196A 8584 8701 8860 9540 9545 10398 10400 10873 11719 11914 12705 13263 13392 14318 14766 14783 15043 15244 15301 15326 15487T 16223 16294 16298 16325 16327
[11]	JX413048	Pehuenche	Chile (Trapa Trapa)	C1b13c1	73 194 249d 258 263 290-291d 309+C 315+C 489 493 750 1438 2706 3552A 4715 4769 7028 7091 7196A 8584 8701 8860 9540 9545 10398 10400 10742 10873 11719 11914 12705 13263 13392 14318 14766 14783 15043 15244 15301 15326 15487T 16223 16298 16325 16327
[12]	KF162561	-	Denmark	C1b13c1	73 152N 249d 258 263 274-302d 310N 489 493 750 1438 1520N 2603N 2604N 2613N 2706N 3552A 4715 4769 5644N 7028 7091 7196A 7579N 7630N 8584 8591-8605d 8614N 8701 8860 9540 9545 10398N 10400 10873 11719 11914 12705 13263 13392 14318 14766 14783 15043 15244 15301 15326 15487T 16223 16294 16298 16325 16327
[11]	JX413044	Mapuche	Argentina (Neuquén)	*C1b13c2*	73 249d 258 263 290-291d 309+C 315+C 489 493 523-524d 750 1438 2706 3552A 4715 4769 5775 7028 7091 7196A 8584 8701 8860 9540 9545 10398 10400 10873 11719 11914 12705 13263 14318 14766 14783 15043 15244 15301 15326 15487T 16223 16298 16325 16327 16519
[11]	JX413045	Rural	Chile (Laitec, Chiloe Island)	*C1b13c2*	73 194 249d 258 263 290-291d 309+C 315+C 489 493 523-524d 750 1346 1438 2706 3552A 4715 4769 5775 7028 7091 7196A 8584 8701 8860 9540 9545 10398 10400 10873 11719 11914 12705 13263 14318 14766 14783 15043 15244 15301 15326 15487T 16223 16298 16325 16327
[11]	JX413049	Urban	Chile (San Felipe, Aconcagua)	C1b13d	73 249d 258 263 290-291d 309+C 315+C 489 493 523-524d 750 1438 2706 3552A 4715 4769 7028 7091 7196A 8584 8701 8860 9540 9545 10398 10400 10873 11117 11719 11914 12705 13263 13965 14318 14766 14783 15043 15301 15326 15487T 16051 16223 16298 16325 16327
[11]	JX413050	Chilean	Chile	C1b13d	73 249d 258 263 290-291d 309+C 315+C 489 493 523-524d 750 1438 2706 3552A 4715 4769 7028 7091 7196A 8584 8701 8860 9540 9545 10398 10400 10873 11719 11914 12705 13263 13674 13965 14318 14766 14783 15043 15301 15326 15487T 16051 16189 16223 16298 16325 16327
[11]	JX413051	Rural	Chile (Quetalmahue, Chiloe Island)	C1b13e	73 249d 258 263 290-291d 309+C 315+C 489 493 523-524d 750 1438 2706 3552A 4715 4769 7028 7091 7196A 8584 8701 8860 9540 9545 10398 10400 10873 11719 11914 12705 13263 14318 14766 14783 14944 15043 15301 15326 15487T 16223 16293 16298 16325 16327
[11]	JX413052	Chilean	Chile	C1b13e	73 249d 258 263 290-291d 309+C 315+C 489 493 523-524d 750 1438 2706 3552A 4715 4769 7028 7091 7196A 7269 8584 8701 8860 9540 9545 10398 10400 10873 11719 11914 12175 12705 13263 14318 14766 14783 14944 15043 15301 15326 15487T 16119 16223 16298 16325 16327
[11]	JX413053	Rural	Chile (Laitec, Chiloe Island)	C1b13e	73 249d 258 263 290-291d 315+C 489 493 523-524d 750 1438 2706 3552A 4715 4769 7028 7091 7196A 8584 8701 8860 9540 9545 10398 10400 10873 11719 11914 12705 13263 14318 14766 14783 14944 15043 15301 15326 15487T 16093 16223 16298 16325 16327
[11]	JX413054	Urban	Chile (Los Andes, Aconcagua)	C1b13e	73 103 150 249d 258 263 290-291d 315+C 489 493 523-524d 750 1438 2706 3552A 4715 4769 7028 7091 7196A 8584 8701 8860 9540 9545 10398 10400 10873 11719 11914 12705 13263 14318 14693 14766 14783 14944 15043 15301 15326 15487T 16223 16298 16325 16327
[9]	HQ012208	Mexica American	Central America	C1b14	73 195 241 249d 263 290-291d 310 315+C 489 493 523-524d 750 1438 2706 3552A 4715 4769 5894 7028 7196A 8047 8584 8701 8860 9540 9545 10397 10398 10400 10873 11719 11914 12705 13263 14318 14766 14783 15043 15301 15326 15487T 16181 16223 16298 16325 16327 16336
[13]	KJ923846	Zapotec	Mexico	C1b14	73 153 215 249d 263 290-291d 315+C 489 493 523-524d 750 1438 2706 3204 3552A 4715 4769 5894 7028 7196A 8584 8701 8860 9540 9545 10040 10397 10398 10400 10873 11719 11914 12705 13263 14318 14766 14783 15043 15301 15326 15487T 16181 16192 16223 16298 16327
taken from 1000Genomes	NA19773	Mexica American	USA (California)	C1b14	73 249d 263 290-291d 315+C 489 493 523-524d 709 750 1438 2706 3552A 4715 4769 5894 6872 7028 7196A 8584 8701 8860 9419 9540 9545 10397 10398 10400 10873 11719 11914 12705 13263 13953 14318 14766 14783 15043 15301 15326 15487T 16172 16181 16223 16298 16325 16327
taken from 1000Genomes	HG02275	-	Peru (Lima)	*C1b15*	73 201 249d 263 290-291d 309+C 315+C 377 489 493 750 1438 2706 2884 3552A 4715 4769 7028 7196A 8584 8701 8860 9540 9545 9861 10398 10400 10586 10873 11719 11914 12705 13263 14318 14766 14783 14983 15043 15301 15326 15487T 16192 16223 16266 16298 16325 16327 16519
[5]	EU095223	Zoró	Brazil? (East)	*C1b15a*	73 114 249d 263 290-291d 309+CC 315+C 489 493 523-524d 750 1438 2706 3552A 4715 4769 7028 7196A 8383 8584 8701 8860 9540 9545 10398 10400 10586 10873 11719 11914 12193 12705 13263 14318 14766 14783 15043 15301 15326 15487T 16093 16223 16256 16298 16325 16327 16519
[5]	EU095224	Zoró	Brazil? (East)	*C1b15a*	73 114 249d 263 290-291d 309+CC 315+C 489 493 523-524d 750 1438 2706 3552A 4715 4769 7028 7196A 8383 8584 8701 8860 9540 9545 10398 10400 10586 10873 11719 11914 12193 12705 13263 14318 14766 14783 15043 15301 15326 15487T 16093 16223 16298 16325 16327 16519

Unpublised data	JX669203	-	Peru (Huancayo)	*C1b16*	73 249d 263 290-291d 309+C 315+C 489 493 523-524d 750 1438 2706 3552A 4715 4769 6629 7028 7196A 8194 8584 8701 8860 9540 9545 10398 10400 10873 11719 11914 12705 13263 14318 14766 14783 15043 15301 15326 15487T 16223 16298 16325 16327 16390 16505
Unpublised data	JX669309	-	Peru (Lima)	*C1b16*	73 249d 263 290-291d 309+CC 315+C 489 493 523-524d 750 1438 2706 3552A 3891 4715 4769 6629 7028 7196A 8400 8584 8701 8860 9540 9545 10398 10400 10873 11719 11914 12705 13263 14318 14766 14783 15043 15301 15326 15487T 16223 16298 16325 16327 16390
Unpublised data	JX669191	-	Peru (Huancayo)	*C1b17*	73 93 204 207 249d 263 290-291d 315+C 489 493 523-524d 750 1438 2706 3552A 4715 4769 7028 7196A 8580 8584 8701 8860 9540 9545 10398 10400 10873 11253 11719 11914 12705 13263 14318 14766 14783 15043 15301 15326 15487T 16156 16223 16298 16325 16327 16519
Unpublised data	JX669329	-	Peru (Lima)	*C1b17*	73 207 249d 263 290-291d 309+C 315+C 489 493 523-524d 750 1438 2706 3552A 4715 4769 7028 7196A 8584 8701 8860 9540 9545 10398 10400 10873 11253 11719 11914 12705 13263 14318 14766 14783 15043 15301 15326 15487T 16111 16156 16223 16298 16325 16327
taken from 1000Genomes	HG01950	-	Peru (Lima)	*C1b18*	73 143 249d 263 290-291d 315+C 470 477 489 493 523-524d 750 1438 2706 3552A 4715 4769 7028 7196A 8584 8701 8860 9540 9545 10398 10400 10873 11719 11914 12242 12561 12705 13263 13547 14318 14560 14687 14766 14783 15043 15301 15326 15487T 16217 16223 16298 16325 16327
Unpublised data	JX669341	-	Peru (Lima)	*C1b18*	73 152 249d 263 290-291d 309+C 315+C 489 493 523-524d 750 1438 2706 3552A 4715 4769 7028 7196A 8584 8701 8860 9540 9545 10398 10400 10873 11719 11914 12705 13263 14318 14569 14687 14766 14783 15043 15301 15326 15487T 16223 16298 16325 16327
taken from 1000Genomes	HG02292	-	Peru (Lima)	*C1b19*	73 249d 263 290-291d 309+C 315+C 489 493 523-524d 750 1438 2706 2977 3552A 4715 4769 7028 7196A 8334 8584 8701 8790 8860 9540 9545 9682 10398 10400 10873 11649 11719 11914 12535 12705 13153 13263 14318 14766 14783 15043 15301 15313 15326 15487T 16215 16223 16298 16325 16327
Unpublised data	JX669250	-	Peru (Lima)	*C1b19*	73 150 188 249d 263 290-291d 309+CC 315+C 489 493 523-524d 750 1438 2706 3552A 4715 4769 7028 7196A 8584 8701 8860 9540 9545 10398 10400 10873 11719 11914 12535 12705 13263 14318 14766 14783 15043 15301 15313 15326 15487T 16170 16223 16298 16325 16327
[4]	EF657584	-	North America	*C1b1a*	750 1438 2706 3552A 4715 4769 7028 7196A 8584 8701 8860 9540 9545 10398 10400 10873 11147 11719 11914 12705 13263 14318 14766 14783 15043 15301 15326 15487T
[9]	HQ012186	Mexica American	Central America	*C1b1a*	73 249d 263 290-291d 309+C 315+C 489 493 523-524d 750 1438 2706 3552A 4715 4769 7028 7196A 8389 8584 8701 8860 9540 9545 10398 10400 10873 11147 11314 11719 11914 12705 13263 14040 14318 14766 14783 15043 15301 15326 15487T 16223 16274 16298 16325 16327 16519
[9]	HQ012193	Mexica American	Central America	*C1b1a*	73 249d 263 290-291d 309+C 315+C 489 493 523-524d 750 1438 2706 3552A 4715 4769 7028 7196A 8392 8584 8701 8860 9540 9545 10398 10400 10873 11147 11314 11719 11914 12705 13263 14040 14318 14766 14783 15043 15301 15326 15487T 16223 16274 16298 16325 16327 16519
[10]	JQ703853	-	-	*C1b1a*	73 249d 263 290-291d 309+C 315+C 489 493 523-524d 750 1438 2706 3552A 4715 4769 7028 7196A 8392 8584 8701 8860 9540 9545 10398 10400 10873 11147 11314 11719 11914 12285 12705 13263 14040 14318 14766 14783 15043 15301 15326 15487T 16223 16274 16298 16325 16327 16519
[2]	AY195759	-	Native American	*C1b1b*	73 249d 263 290-291d 309+C 315+C 489 493 523-524d 750 1438 2706 3552A 4715 4769 5493 6962 7028 7196A 8555 8584 8701 8860 9540 9545 10151 10398 10400 10873 11147 11719 11914 12705 13263 14318 14766 14783 15043 15301 15326 15487T 16223 16298 16325 16327 16519
[9]	HQ012194	Mexica American	Central America	*C1b1b*	73 249d 263 290-291d 309+C 315+C 489 493 523-524d 750 1438 2706 3552A 4715 4769 5493 7028 7196A 8555 8584 8701 8860 9540 9545 10398 10400 10873 11147 11719 11914 12705 13263 14318 14766 14783 15043 15301 15326 15487T 16129 16223 16224 16298 16325 16327 16519
[1]	AF382009	-	Spain (Canary Islands)	C1b2	73 249d 290-291d 315+C 489 493 523-524d 750 1438 2706 3552A 4715 4769 7013 7028 7196A 8584 8701 8860 9540 9545 9557 10398 10400 10873 11719 11914 12454 12574 12705 13263 14318 14766 14783 15043 15301 15326 15487T 16223 16298 16325 16327 16519
[3]	DQ282447	Hispanic	USA	C1b2	73 249d 290-291d 309+C 315+C 489 493 523-524d 750 1438 2706 3552A 4242 4715 4769 7013 7028 7196A 8584 8701 8860 9540 9545 9557 10398 10400 10873 11719 11914 12454 12705 13263 14318 14766 14783 15043 15301 15326 15487T 16223 16298 16325 16327 16519
[3]	DQ282448	Hispanic	USA	C1b2	73 249d 290-291d 315+C 489 493 523-524d 750 1438 2706 3552A 4242 4715 4769 7013 7028 7196A 8584 8701 8860 9540 9545 9557 10398 10400 10873 11719 11914 12454 12705 13263 14318 14766 14783 15043 15301 15326 15487T 16223 16298 16325 16327 16519
[3]	DQ282449	Hispanic	USA	C1b2	73 249d 290-291d 315+C 489 493 523-524d 750 1438 2706 3552A 4242 4715 4769 7013 7028 7196A 8584 8701 8860 9540 9545 9557 10398 10400 10873 11719 11914 12454 12705 13263 14318 14766 14783 14883 15043 15301 15326 15487T 16223 16298 16325 16327 16519
[3]	DQ282450	Hispanic	USA	C1b2	73 249d 290-291d 315+C 489 493 523-524d 750 1438 2706 3552A 4242 4715 4769 7013 7028 7196A 8584 8701 8860 9540 9545 9557 10398 10400 10873 11719 11914 12454 12705 13263 14318 14766 14783 15043 15301 15326 15487T 16223 16298 16325 16327 16519
[3]	DQ282451	Hispanic	USA	C1b2	73 249d 290-291d 315+C 489 493 523-524d 750 1438 2706 3552A 4242 4715 4769 7013 7028 7196A 8584 8701 8860 9540 9545 9557 10398 10400 10873 11719 11914 12454 12705 13263 14318 14766 14783 15043 15301 15326 15487T 16223 16298 16325 16327 16519
[3]	DQ282452	Hispanic	USA	C1b2	73 249d 290-291d 315+C 489 493 523-524d 750 1438 2706 3552A 4242 4715 4769 7013 7028 7196A 8584 8701 8860 9540 9545 9557 10398 10400 10873 11719 11914 12454 12705 13263 14318 14766 14783 15043 15301 15326 15487T 16223 16298 16325 16327 16519
[3]	DQ282453	Hispanic	USA	C1b2	73 249d 290-291d 315+C 489 493 523-524d 750 1438 2706 3552A 4242 4715 4769 7013 7028 7196A 8584 8701 8860 9540 9545 9557 10398 10400 10873 11719 11914 12454 12705 13263 14318 14766 14783 15043 15301 15326 15487T 16223Y 16298 16325 16327 16519
[3]	DQ282454	Hispanic	USA	C1b2	73 249d 290-291d 315+C 489 493 523-524d 750 1438 2706 3552A 4242 4715 4769 7013 7028 7196A 8584 8701 8860 9540 9545 9557 10398 10400 10873 11719 11914 12454 12705 13263 14318 14766 14783 15043 15301 15326 15487T 16223 16298 16325 16327 16519
[3]	DQ282455	Hispanic	USA	C1b2	73 249d 290-291d 315+C 489 493 523-524d 750 1438 2706 3552A 4242 4715 4769 7013 7028 7196A 8584 8701 8860 9540 9545 9557 10398 10400 10873 11719 11914 12454 12705 13263 14318 14766 14783 15043 15301 15326 15487T 16223 16298 16325 16327 16519
[3]	DQ282456	Hispanic	USA	C1b2	73 249d 290-291d 315+C 489 493 523-524d 750 1438 2706 3552A 4242 4715 4769 7013 7028 7196A 8584 8701 8860 9540 9545 9557 10398 10400 10873 11719 11914 12454 12705 13263 14318 14766 14783 15043 15301 15326 15487T 16223 16298 16325 16327 16519
[3]	DQ282457	Hispanic	USA	C1b2	73 249d 290-291d 315+C 489 493 523-524d 750 1438 2706 3552A 4242 4715 4769 7013 7028 7196A 8584 8701 8860 9540 9545 9557 10398 10400 10873 11719 11914 12454 12705 13263 14318 14766 14783 15043 15301 15326 15487T 16223 16298 16325 16327 16519
[3]	DQ282458	Hispanic	USA	C1b2	73 249d 290-291d 315+C 489 493 523-524d 750 1438 2706 3552A 4242 4715 4769 7013 7028 7196A 8584 8701 8860 9540 9545 9557 10398 10400 10873 11719 11914 12454 12705 13263 14318 14766 14783 15043 15301 15326 15487T 16223 16298 16325 16327 16519

[4]	EF657282	-	North America	C1b2	750 1438 2706 3552A 4242 4715 4769 7013 7028 7196A 8584 8701 8860 9540 9545 9557 10398 10400 10873 11719 11914 12454 12705 13263 14318 14766 14783 15043 15301 15326 15487T
Family tree	GQ397486	Puerto Rican	Puerto Rico	C1b2	73 249d 290-291d 315+C 489 493 523-524d 750 1438 2706 3552A 4242 4715 4769 7013 7028 7196A 8584 8701 8860 9540 9545 9557 10398 10400 10873 11719 11914 12454 12705 13263 14318 14766 14783 15043 15301 15326 15487T 16223 16298 16325 16327 16519
taken from 1000Genomes	HG00641	Puerto Rican	Puerto Rico	C1b2	73 204 249d 290-291d 315+C 489 493 523-524d 750 1438 2706 3552A 4242 4715 4769 7013 7028 7196A 8584 8701 8860 9540 9545 9557 10398 10400 10873 11719 11914 12454 12705 13263 14318 14766 14783 15043 15301 15326 15431 15487T 16223 16298 16325 16327 16519
taken from 1000Genomes	HG00734	Puerto Rican	Puerto Rico	C1b2	73 249d 290-291d 315+C 489 493 523-524d 750 1438 2706 3552A 4242 4715 4769 7013 7028 7196A 8584 8701 8860 9540 9545 9557 10398 10400 10549 10873 11719 11914 12454 12705 13263 14053 14318 14518 14766 14783 15043 15301 15326 15487T 16223 16298 16325 16327 16519
taken from 1000Genomes	HG00737	Puerto Rican	Puerto Rico	C1b2	73 249d 290-291d 315+C 489 493 523-524d 750 1438 2706 3552A 4242 4715 4769 7013 7028 7196A 8584 8701 8860 9309 9540 9545 9557 10398 10400 10873 11719 11914 12454 12705 13263 14318 14766 14783 15043 15301 15326 15487T 16223 16298 16325 16327 16519
taken from 1000Genomes	HG00742	Puerto Rican	Puerto Rico	C1b2	73 249d 290-291d 315+C 489 493 523-524d 750 1438 2706 3552A 4242 4715 4769 4976 7013 7028 7196A 8584 8701 8860 9540 9545 9557 10398 10400 10873 11719 11914 12454 12705 13263 14318 14766 14783 15043 15301 15326 15487T 16209 16223 16298 16325 16327 16519
taken from 1000Genomes	HG00743	Puerto Rican	Puerto Rico	C1b2	73 249d 290-291d 315+C 489 493 523-524d 750 1438 2706 3552A 4242 4715 4769 7013 7028 7196A 8584 8701 8860 9540 9545 9557 10398 10400 10873 11719 11914 12454 12705 13263 14318 14766 14783 15043 15301 15326 15487T 16223 16298 16325 16327 16519
taken from 1000Genomes	HG01048	Puerto Rican	Puerto Rico	C1b2	73 249d 290-291d 309+C 315+C 489 493 523-524d 750 1438 2706 3552A 4242 4715 4769 7013 7028 7196A 8584 8701 8860 9540 9545 9557 10398 10400 10873 11719 11914 12454 12705 13263 14318 14766 14783 15043 15301 15326 15487T 16065 16223 16298 16325 16327 16519
taken from 1000Genomes	HG01058	Puerto Rican	Puerto Rico	C1b2	73 249d 290-291d 315+C 489 493 523-524d 750 1438 2706 3552A 4242 4715 4769 7013 7028 7196A 8584 8701 8860 9540 9545 9557 10398 10400 10873 11719 11914 12454 12705 13263 14318 14766 14783 15043 15301 15326 15487T 16223 16298 16325 16327 16519
taken from 1000Genomes	HG01067	Puerto Rican	Puerto Rico	C1b2	73 249d 290-291d 315+C 489 493 523-524d 750 1438 2706 3552A 4242 4715 4769 7013 7028 7196A 8584 8701 8860 9540 9545 9557 10398 10400 10873 11719 11914 12454 12705 13263 14318 14766 14783 15043 15301 15326 15487T 16223 16298 16325 16327 16519
taken from 1000Genomes	HG01069	Puerto Rican	Puerto Rico	C1b2	73 249d 290-291d 315+C 489 493 523-524d 750 1438 2706 3552A 4242 4715 4769 7013 7028 7196A 8584 8701 8860 9540 9545 9557 10398 10400 10873 11719 11914 12454 12612 12705 13263 14318 14766 14783 15043 15301 15326 15487T 16223 16298 16325 16327 16519
taken from 1000Genomes	HG01072	Puerto Rican	Puerto Rico	C1b2	73 249d 290-291d 315+C 489 493 523-524d 750 1438 2706 3552A 4242 4715 4769 6419C 7013 7028 7196A 8584 8701 8860 9540 9545 9557 10398 10400 10873 11719 11914 12454 12705 13263 14318 14766 14783 15043 15301 15326 15487T 16223 16298 16325 16327 16519
taken from 1000Genomes	HG01092	Puerto Rican	Puerto Rico	C1b2	73 189 249d 290-291d 315+C 489 493 523-524d 710 750 1438 2706 3552A 4242 4715 4769 7013 7028 7196A 8584 8701 8860 9540 9545 9557 10398 10400 10873 11719 11914 12454 12705 13263 14318 14766 14783 15043 15301 15326 15487T 16223 16298 16325 16327 16519
taken from 1000Genomes	HG01102	Puerto Rican	Puerto Rico	C1b2	73 249d 290-291d 315+C 489 493 523-524d 750 1438 2706 3552A 4242 4715 4769 7013 7028 7196A 8584 8701 8860 9540 9545 9557 10398 10400 10873 11719 11914 12454 12705 13263 14318 14766 14783 15043 15301 15326 15487T 16223 16298 16325 16327 16519
taken from 1000Genomes	HG01190	Puerto Rican	Puerto Rico	C1b2	73 249d 290-291d 315+C 489 493 523-524d 750 1438 2706 3552A 4242 4715 4769 7013 7028 7196A 8584 8701 8860 9540 9545 9557 10398 10400 10873 11719 11914 12454 12705 13263 14318 14766 14783 15043 15301 15326 15487T 16223 16298 16325 16327 16519
taken from 1000Genomes	HG01204	Puerto Rican	Puerto Rico	C1b2	73 249d 290-291d 315+C 489 493 523-524d 750 1438 2706 3552A 4242 4715 4769 7013 7028 7196A 8584 8701 8860 9540 9545 9557 10398 10400 10873 11719 11914 12454 12705 13263 14318 14766 14783 15043 15301 15326 15487T 16223 16298 16325 16327 16519
taken from 1000Genomes	HG01312	Puerto Rican	Puerto Rico	C1b2	73 249d 290-291d 315+C 489 493 523-524d 750 1438 2706 3552A 4242 4715 4769 7013 7028 7196A 8584 8701 8860 9540 9545 9557 10398 10400 10873 11719 11914 12454 12705 13263 14318 14766 14783 15043 15301 15326 15487T 16223 16298 16325 16327 16519
taken from 1000Genomes	HG01392	Puerto Rican	Puerto Rico	C1b2	73 249d 290-291d 315+C 489 493 523-524d 750 1438 2706 3552A 4242 4715 4769 7013 7028 7196A 8584 8701 8860 9540 9545 9557 10398 10400 10873 11719 11914 12454 12705 13263 14318 14766 14783 15043 15301 15326 15487T 16223 16298 16325 16327 16519
[10]	JN546585	-	-	C1b2	73 249d 290-291d 315+C 489 493 523-524d 750 1438 2706 3552A 4242 4715 4769 7013 7028 7196A 8584 8701 8860 9165 9540 9545 9557 10398 10400 10873 11719 11914 12454 12705 13263 14318 14766 14783 15043 15301 15326 15487T 16223 16298 16325 16327 16519
[10]	JQ701820	-	-	C1b2	73 152Y 249d 290-291d 315+C 489 493 523-524d 750 1438 2706 3552A 4242 4715 4769 7013 7028 7196A 8584 8701 8860 9540 9545 9557 10398 10400 10873 11719 11914 12454 12705 13263 14318 14766 14783 15043 15301 15326 15487T 16223 16298 16325 16327 16519
taken from 1000Genomes	HG01947	-	Peru (Lima)	*C1b20*	73 249d 263 290-291d 309+C 315+C 489 493 523-524d 750 1438 2706 3552A 4715 4769 7028 7196A 8281-8289d 8584 8610 8701 8860 9540 9545 9692 10398 10400 10873 11719 11914 12112 12705 13263 13752 14318 14766 14783 15043 15301 15326 15487T 16223 16298 16325 16327 16519
taken from 1000Genomes	HG02266	-	Peru (Lima)	*C1b20*	73 182 249d 263 290-291d 309+C 315+C 489 493 523-524d 573+CC 750 1438 2706 3552A 4715 4769 7028 7196A 8281-8289d 8584 8701 8860 9540 9545 9692 10398 10400 10873 11719 11914 12705 13263 13752 14318 14766 14783 15043 15301 15326 15487T 16220 16223 16298 16325 16327 16399 16519
Unpublised data	JX669365	-	Peru (Lima)	*C1b21*	73 249d 263 290-291d 315+C 489 493 523-524d 750 1438 2706 3552A 4715 4769 5821 6605 7028 7196A 8584 8701 8860 9540 9545 10398 10400 10873 10993 11719 11914 12705 13263 13602 14318 14766 14783 15043 15244 15301 15326 15487T 16094 16223 16298 16325 16327 16362
Unpublised data	JX669288	-	Peru (Lima)	*C1b21a*	73 249d 263 290-291d 309+C 315+C 489 493 523-524d 636 750 1438 2706 3552A 4715 4769 5821 7028 7196A 8155 8584 8701 8860 9540 9545 10398 10400 10873 11719 11914 12705 13263 14318 14766 14783 15043 15301 15326 15487T 16223 16298 16325 16327 16362 16519
Unpublised data	JX669377	-	Peru (Lima)	*C1b21a*	64 73 249d 263 290-291d 309+C 315+C 319 489 493 636 750 1438 2706 3552A 4715 4769 5441 5821 7028 7196A 8584 8701 8860 9540 9545 10398 10400 10873 11719 11779 11914 12705 13263 14318 14766 14783 14832 15043 15301 15326 15487T 16048R 16193 16223 16298 16325 16327 16348
taken from 1000Genomes	HG01942	-	Peru (Lima)	*C1b22*	73 76 78 79T 198 249d 263 290-291d 309+CC 315+C 489 493 523-524d 750 1438 2706 3552A 4715 4769 5894+C 6997 7028 7196A 7521 8584 8701 8860 9540 9545 10398 10400 10873 11719 11914 12705 12771 13263 13708 14016 14318 14766 14783 15043 15301 15326 15487T 16093 16223 16240 16298 16325 16327
Unpublised data	JX669398	-	Peru (Lima)	*C1b22*	73 76 79T 249d 263 290-291d 315+C 489 493 523-524d 573+XC 750 1438 2706 3552A 4715 4769 7028 7196A 8584 8701 8860 9540 9545 10398 10400 10873 11719 11914 12705 13263 13708 14224 14318 14766 14783 15043 15301 15326 15487T 15643 16103 16209 16223 16256 16298 16325 16327

Unpublised data	JX669162	-	Peru (Cajamarca)	*C1b2a*	73 249d 263 290-291d 309+C 315+C 489 493 523-524d 750 1438 2706 3552A 4242 4715 4769 7013 7028 7196A 7841T 8584 8701 8860 9540 9545 9557 10398 10400 10873 11719 11914 12651 12705 13263 14067Y 14318 14766 14783 15043 15301 15326 15487T 16223 16298 16319 16325 16327
Unpublised data	JX669406	-	Peru (Ucayali)	*C1b2a*	73 249d 263 290-291d 309+C 315+C 489 493 750 1438 2706 3552A 3579 4242 4715 4769 7013 7028 7196A 8584 8701 8860 9540 9545 9557 9914 10398 10400 10873 11719 11914 12705 13135 13263 14318 14766 14783 15043 15301 15326 15487T 16223 16298 16325 16327
[3]	DQ282464	Hispanic	USA	C1b3	73 249d 263 290-291d 315+C 489 493 523-524d 750 1438 2706 3552A 4715 4769 5664 6293 7028 7196A 8584 8701 8860 9540 9545 10398 10400 10873 11719 11914 12630 12705 13263 14318 14766 14783 15043 15301 15326 15487T 16223 16298 16325 16327 16519
Unpublised data	JX669316	-	Peru (Lima)	C1b3	73 201 249d 263 290-291d 315+C 489 493 523-524d 750 1438 2706 3204 3552A 4715 4769 7028 7196A 8584 8701 8860 9115 9540 9545 9644 10398 10400 10454 10873 11719 11914 12346 12630 12705 13263 14318 14766 14783 15043 15301 15326 15487T 16093 16192 16223 16298 16311 16325 16327
taken from 1000Genomes	NA19788	Mexica American	USA (California)	C1b3	73 203 204 249d 263 290-291d 315+C 489 493 523-524d 750 1438 2706 3552A 4715 4769 7028 7196A 8567 8584 8701 8860 9540 9545 10398 10400 10873 11719 11914 12630 12705 13263 14318 14750 14766 14783 15043 15301 15326 15487T 16126 16223 16298 16325 16327 16519
[3]	DQ282475	Hispanic	USA	C1b4	73 143 249d 263 290-291d 309+C 315+C 489 493 523-524d 750 2706 3552A 4167 4715 4769 7028 7196A 8584 8701 8860 9530 9540 9545 10398 10400 10873 11719 11914 12705 13263 14318 14524 14766 14783 15043 15301 15326 15487T 16086 16183C 16189 16193+N 16223 16278 16298 16325 16327
[3]	DQ282476	Hispanic	USA	C1b4	73 143 249d 263 290-291d 309+CN 315+C 489 493 523-524d 750 2706 3552A 4167 4715 4769 7028 7196A 8584 8701 8860 9540 9545 10398 10400 10873 11719 11914 12705 13263 14318 14524 14766 14783 15043 15301 15326 15487T 16086 16183C 16189 16193+N 16223 16278 16298 16325 16327
[7]	EU431085	-	North America	C1b4	73 143 249d 263 290-291d 309+C 315+C 489 493 523-524d 750 2706 3552A 4167 4715 4769 7028 7196A 8584 8701 8860 9540 9545 10398 10400 10873 11719 11914 12705 13263 14318 14524 14766 14783 15043 15301 15326 15487T 16086 16183C 16189 16223 16278 16298 16325 16327
taken from 1000Genomes	HG01070	Puerto Rican	Puerto Rico	C1b4	73 143 249d 263 290-291d 309+C 315+C 489 493 523-524d 750 2706 3552A 4167 4715 4769 7028 7196A 8584 8701 8860 9540 9545 10398 10400 10873 11719 11914 12705 13263 14318 14524 14766 14783 15043 15301 15326 15487T 16086 16182+C 16183C 16189 16223 16278 16298 16325 16327
taken from 1000Genomes	HG01086	Puerto Rican	Puerto Rico	C1b4	73 143 249d 263 290-291d 309+C 315+C 489 493 523-524d 750 2706 3552A 4167 4715 4769 7028 7196A 8584 8701 8860 9540 9545 10310 10398 10400 10873 11719 11914 12705 13263 14318 14524 14766 14783 15043 15301 15326 15487T 15978 16086 16183C 16189 16193d 16223 16278 16298 16325 16327
taken from 1000Genomes	HG01089	Puerto Rican	Puerto Rico	C1b4	73 143 152 249d 263 290-291d 309+C 315+C 489 493 523-524d 750 2706 3552A 4167 4715 4769 7028 7196A 8584 8701 8860 9540 9545 10398 10400 10873 11719 11914 12705 13263 14318 14524 14766 14783 15043 15301 15326 15487T 16086 16182+C 16183C 16189 16223 16278 16298 16325 16327
taken from 1000Genomes	HG01101	Puerto Rican	Puerto Rico	C1b4	73 143 249d 263 290-291d 315+C 489 493 523-524d 750 2706 3552A 4167 4715 4769 7028 7196A 8584 8701 8860 9540 9545 10398 10400 10873 11719 11914 12705 13263 14318 14524 14766 14783 15043 15301 15326 15487T 16086 16182+C 16183C 16189 16223 16278 16298 16325 16327
taken from 1000Genomes	HG01396	Puerto Rican	Puerto Rico	C1b4	73 143 249d 263 290-291d 309+C 315+C 489 493 523-524d 750 2706 3552A 4167 4715 4769 7028 7196A 8584 8701 8860 9540 9545 10398 10400 10873 11719 11914 12705 13263 14318 14524 14766 14783 15043 15301 15326 15487T 16086 16182+C 16183C 16189 16223 16278 16298 16325 16327
taken from 1000Genomes	HG01402	Puerto Rican	Puerto Rico	C1b4	73 143 249d 263 290-291d 309+CC 315+C 489 493 523-524d 750 1031 2706 3552A 4167 4715 4769 7028 7196A 8584 8701 8860 9540 9545 10398 10400 10873 11719 11914 12705 13263 14318 14524 14766 14783 15043 15301 15326 15487T 16086 16182+C 16183C 16189 16223 16278 16298 16325 16327
[10]	JQ705585	-	-	C1b4	73 143 152 249d 263 290-291d 309+C 315+C 489 493 523-524d 750 2706 3552A 4167 4715 4769 7028 7196A 8584 8701 8860 9540 9545 10398 10400 10873 11719 11914 12705 13263 14318 14524 14766 14783 15043 15301 15326 15487T 16086 16183C 16189 16223 16278 16298 16325 16327
[3]	DQ282461	Hispanic	USA	C1b5a	73 249d 263 290-291d 309+CN 315+C 489 493 523-524d 750 1005 1438 2706 3552A 4715 4769 5605 7028 7196A 8584 8701 8860 9540 9545 10398 10400 10873 11719 11914 11989 12705 13263 14318 14766 14783 15043 15119 15301 15326 15487T 16223 16298 16325 16327 16519
[3]	DQ282469	Hispanic	USA	C1b5a	73 249d 263 290-291d 309+N 315+C 489 493 523-524d 750 1005 1438 2706 3357T 3552A 4715 4769 7028 7196A 7628A 8584 8701 8860 9540 9545 10398 10400 10873 11719 11914 11989 12705 13263 14318 14766 14783 15043 15119 15301 15326 15487T 15790 16223 16298 16325 16327
[9]	HQ012213	Mexica American	Central America	C1b5a	73 146 249d 263 290-291d 315+C 489 493 523-524d 750 1005 1438 2706 3552A 4715 4769 7028 7196A 8584 8701 8860 9540 9545 10398 10400 10873 11719 11914 11989 12705 13263 14318 14766 14783 15043 15119 15301 15326 15487T 16223 16298 16325 16327
[10]	JQ705943	-	-	C1b5a	73 249d 263 290-291d 309+C 315+C 489 493 523-524d 750 1005 1438 2706 3531 3552A 4715 4769 5821 7028 7196A 7747 8572 8584 8701 8860 9540 9545 10398 10400 10873 11719 11914 11989 12705 13263 14318 14766 14783 15043 15119 15301 15326 15487T 16223 16298 16320Y 16325 16327 16519
[9]	HQ012205	Mexica American	Central America	C1b5b	73 249d 263 290-291d 309+C 315+C 489 493 523-524d 750 1438 2706 3552A 4715 4769 5157 7028 7196A 8584 8701 8860 9540 9545 10398 10400 10873 11719 11914 11989 12705 13263 14318 14766 14783 15043 15119 15301 15326 15487T 16223 16249 16298 16325 16327 16354
[10]	JQ702534	-	-	C1b5b	73 249d 255 263 290-291d 315+C 331 489 493 523-524d 750 1438 2706 3552A 4715 4769 5157 7028 7196A 8584 8701 8860 9540 9545 10398 10400 10873 11719 11914 11989 12705 13263 14061 14070 14318 14766 14783 15043 15119 15301 15326 15487T 15586 16223 16249 16298 16325 16327
[10]	JQ702595	-	-	C1b5b	73 249d 263 290-291d 315+C 489 493 523-524d 750 1438 2706 3552A 4715 4769 5157 7028 7196A 8584 8701 8860 9449 9540 9545 10398 10400 10873 11719 11755 11914 11989 12705 13263 14318 14766 14783 15043 15119 15301 15326 15487T 16223 16249 16298 16325 16327
[10]	JQ705570	-	-	C1b5b	73 249d 255 263 290-291d 315+C 331 489 493 523-524d 750 1438 2706 3552A 4715 4769 5157 7028 7196A 8584 8701 8860 9540 9545 10398 10400 10873 11719R 11914 11989 12705 13263 14061 14070 14318 14766 14783 15043 15119 15301 15326 15487T 15586 16223 16249 16298 16325 16327
[5]	EU095229	Yanomama	Brazil?	C1b6	73 249d 263 290-291d 309+C 315+C 489 493 523-524d 750 1438 2706 3552A 4715 4769 7028 7196A 8584 8701 8848 8860 9540 9545 10398 10400 10873 11719 11914 12705 13263 13326 14318 14766 14783 15043 15301 15326 15487T 16223 16271 16298 16325 16327 16357 16519
[5]	EU095230	Yanomama	Brazil?	C1b6	73 249d 263 290-291d 309+C 315+C 441G 489 493 523-524d 568N 750 1438 2706 3552A 4715 4769 7028 7196A 8584 8701 8848 8860 9540 9545 10398 10400 10873 11719 11914 12705 13263 13326 14318 14766 14783 15043 15301 15326 15487T 16223 16271 16298 16325 16327 16357 16519
[5]	EU095231	Yanomama	Brazil?	C1b6	73 249d 263 290-291d 315+C 489 493 523-524d 750 1438 2706 3552A 4715 4769 7028 7196A 8584 8701 8848 8860 9540 9545 10310 10398 10400 10873 11719 11914 12705 13263 13326 14318 14766 14783 15043 15301 15326 15487T 16223 16271 16298 16325 16327 16357 16519
[9]	HQ012207	Mexica American	Central America	C1b7	73 143 195 249d 263 290-291d 309+C 315+C 489 493 523-524d 709 750 1438 2706 3552A 4715 4769 5279 7028 7196A 8584 8701 8860 9540 9545 10398 10400 10873 11719 11914 12361 12603 12705 13263 13635 14318 14766 14783 15043 15301 15326 15487T 16223 16298 16311 16325 16327

taken from 1000Genomes	NA19762	Mexica American	USA (California)	C1b7a	73 195 249d 263 290-291d 309+CC 315+C 489 493 523-524d 750 1438 2706 3552A 4715 4769 7028 7196A 8584 8701 8860 9540 9545 10398 10400 10873 11719 11914 12705 13263 13635 14318 14766 14783 15043 15301 15326 15470 15487T 16223 16298 16311 16325 16327 16519
[9]	HQ012195	Mexica American	Central America	*C1b7a1*	60+T 64 73 249d 263 290-291d 309+C 315+C 489 493 523-524d 750 1310 1438 2706 3552A 4715 4769 7028 7196A 8251 8584 8701 8860 9540 9545 10398 10400 10873 11719 11914 12705 13263 13635 14318 14766 14783 15043 15301 15326 15470 15487T 16223 16298 16311 16325 16327 16390
[9]	HQ012215	Mexica American	Central America	*C1b7a1*	73 249d 263 290-291d 309+C 315+C 333 489 493 523-524d 750 1310 1438 2706 3552A 4715 4769 7028 7196A 8584 8701 8860 9540 9545 10398 10400 10873 11719 11914 12705 13263 13635 14318 14766 14783 15043 15301 15326 15470 15487T 16223 16298 16311 16325 16327
[9]	HQ012216	Mexica American	Central America	*C1b7a1*	60 73 249d 263 290-291d 309+CC 315+C 489 493 523-524d 750 1117 1310 1438 2706 3552A 4715 4769 7028 7196A 8584 8701 8860 9540 9545 10398 10400 10873 11719 11914 12705 13263 13635 14318 14766 14783 15043 15301 15326 15470 15487T 16189 16223 16298 16311 16325 16327 16463
[9]	HQ012192	Mexica American	Central America	*C1b7b*	73 195 249d 263 290-291d 309+C 315+C 489 493 523-524d 750 1438 2706 3552A 4715 4769 7028 7196A 7606 8584 8701 8860 9540 9545 10398 10400 10873 11719 11914 12705 13263 13635 14318 14766 14783 15043 15300 15301 15326 15487T 16172 16223 16298 16311 16325 16327 16468
[9]	HQ012196	Mexica American	Central America	*C1b7b*	73 249d 263 290-291d 309+C 315+C 489 493 523-524d 750 1438 2706 3552A 4715 4769 7028 7196A 7606 8584 8701 8860 9540 9545 10398 10400 10873 11719 11914 12705 13263 13635 14318 14389 14766 14783 15043 15301 15326 15487T 16172 16223 16298 16311 16325 16327
[9]	HQ012236	Mexica American	Central America	C1b8	73 195 249d 263 290-291d 309+C 315+C 489 523-524d 750 1438 2706 3552A 4715 4769 5563 7028 7196A 8389 8584 8701 8860 9540 9545 10398 10400 10601 10873 11719 11914 12631G 12705 13263 14318 14766 14783 15043 15301 15326 15487T 15613 16172 16188 16195 16223 16298 16325 16327 16362 16519
[10]	JQ702651	-	-	C1b8	73 143 195 249d 263 290-291d 309+C 315+C 489 493 523-524d 750 1438 2706 3552A 4715 4769 5563 7028 7196A 8389 8584 8701 8860 9540 9545 10398 10400 10601 10873 11719 11914 12631G 12705 13263 14318 14766 14783 15043 15301 15326 15487T 15613 16172 16188 16195 16223 16298 16325 16327 16362 16519
[9]	HQ012210	Mexica American	Central America	C1b8a	73 150 249d 263 290-291d 309+C 315+C 489 493 523-524d 750 1438 2706 3552A 3736 4381 4715 4769 4911G 6260 7028 7196A 8584 8701 8860 9130 9540 9545 10398 10400 10873 11719 11812 11914 12705 13263 14318 14766 14783 15043 15301 15326 15487T 15613 16223 16298 16325 16327 16362
[9]	HQ012201	Mexica American	Central America	*C1b8a1*	73 249d 263 290-291d 309+CC 315+C 489 493 523-524d 750 1438 2706 3552A 3736 4381 4715 4769 4911G 6473 7028 7196A 8701 8860 9130 9540 9545 10398 10400 10873 11719 11812 11914 12705 13263 14318 14766 14783 14813 15043 15301 15326 15487T 15613 16223 16298 16325 16327
[9]	HQ012202	Mexica American	Central America	*C1b8a1*	73 249d 263 290-291d 309+CC 315+C 489 493 523-524d 750 1438 2706 3552A 3736 4381 4715 4769 4911G 6473 7028 7196A 8584 8701 8860 9130 9540 9545 10398 10400 10873 11719 11812 11914 12705 13263 14318 14766 14783 15043 15301 15326 15487T 15613 16223 16298 16325 16327
[9]	HQ012187	Mexica American	Central America	C1b9	73 198 249d 263 290-291d 309+C 315+C 489 493 523-524d 750 1438 2706 3552A 4715 4769 6297 7028 7196A 8047 8584 8701 8860 9540 9545 10398 10400 10873 11719 11914 12705 13263 14318 14766 14783 15043 15301 15326 15487T 16172 16183C 16223 16298 16325 16327
[9]	HQ012190	Mexica American	Central America	C1b9	73 198 249d 263 290-291d 309+C 315+C 489 493 523-524d 750 1438 2706 3552A 4715 4769 5237 6297 7028 7196A 8047 8584 8701 8860 9540 9545 10398 10400 10873 11719 11914 12705 13263 14318 14562G 14766 14783 15043 15301 15326 15487T 16223 16298 16325 16327
[9]	HQ012191	Mexica American	Central America	C1b9	73 198 249d 263 290-291d 309+C 315+C 489 493 523-524d 750 1438 2706 3552A 4715 4769 6297 7028 7196A 8047 8584 8701 8860 9540 9545 10398 10400 10873 11440 11719 11914 12705 13263 14318 14766 14783 15043 15301 15326 15487T 16223 16298 16325 16327
[9]	HQ012199	Mexica American	Central America	C1b9	73 198 249d 263 290-291d 309+C 315+C 489 493 750 1438 2706 3552A 4715 4769 6297 7028 7196A 8047 8584 8701 8860 9540 9545 10398 10400 10873 11719 11914 11944 12705 13263 14226 14318 14766 14783 15043 15301 15326 15487T 16223 16298 16325 16327
[9]	HQ012204	Mexica American	Central America	C1b9	73 198 249d 263 290-291d 309+C 315+C 489 493 750 1438 2706 3552A 4715 4769 6297 7028 7196A 8047 8584 8701 8860 9540 10398 10400 10873 11719 11914 12705 13263 14318 14766 14783 15043 15301 15326 15487T 16223 16298 16325 16327
[9]	HQ012212	Mexica American	Central America	C1b9	73 198 249d 263 290-291d 309+C 315+C 489 493 523-524d 750 1438 2706 3552A 4715 4769 6297 7028 7196A 8047 8584 8701 8860 9540 9545 10398 10400 10873 11719 11914 12705 13263 14318 14766 14783 15043 15301 15326 15487T 16223 16298 16325 16327
[10]	JQ702984	-	-	C1b9	73 146 249d 263 290-291d 309+C 315+C 489 493 523-524d 750 1438 1719 2706 3552A 4715 4769 6297 7028 7196A 8047 8584 8701 8860 9540 9545 10398 10400 10873 11719 11914 12705 13263 14318 14766 14783 15043 15301 15326 15487T 16179 16223 16298 16325 16327

References

[1] Maca-Meyer, N., Gonzalez, A.M., Larruga, J.M., Flores, C. & Cabrera, V.M. Major genomic mitochondrial lineages delineate early human expansions. *BMC Genet.* 2, 13. Epub 2001 Aug 13. (2001).
[2] Mishmar, D. *et al.* Natural selection shaped regional mtDNA variation in humans. *Proc Natl Acad Sci U S A* 100, 171-6 (2003).
[3] Just, R.S., Diegoli, T.M., Saunier, J.L., Irwin, J.A. & Parsons, T.J. Complete mitochondrial genome sequences for 265 African American and U.S. "Hispanic" individuals. *Forensic Sci Int Genet* 2, e45-8 (2008).
[4] Herrnstadt, C. *et al.* Reduced-median-network analysis of complete mitochondrial DNA coding-region sequences for the major African, Asian, and European haplogroups. *Am J Hum Genet.* 70, 1152-71. Epub 2002 Apr 5. (2002).
[5] Fagundes, N.J. *et al.* Mitochondrial population genomics supports a single pre-Clovis origin with a coastal route for the peopling of the Americas. *Am J Hum Genet.* 82, 583-92. Epub 2008 Feb 28. (2008).
[6] Tamm, E. *et al.* Beringian standstill and spread of Native American founders. *PLoS One.* 2, e829. (2007).
[7] Achilli, A. *et al.* The phylogeny of the four pan-American MtDNA haplogroups: implications for evolutionary and disease studies. *PLoS One* 3, e1764 (2008).
[8] Hartmann, A. *et al.* Validation of microarray-based resequencing of 93 worldwide mitochondrial genomes. *Human Mutation* 30, 115-122 (2009).
[9] Kumar, S. *et al.* Large scale mitochondrial sequencing in Mexican Americans suggests a reappraisal of Native American origins. *BMC Evol Biol* 11, 293 (2011).
[10] Behar, D.M. *et al.* A "Copernican" reassessment of the human mitochondrial DNA tree from its root. *Am J Hum Genet* 90, 675-84 (2012).
[11] de Saint Pierre, M. *et al.* Arrival of Paleo-Indians to the southern cone of South America: new clues from mitogenomes. *PLoS One* 7, e51311 (2012).
[12] Li, S. *et al.* Variation and association to diabetes in 2000 full mtDNA sequences mined from an exome study in a Danish population. *Eur J Hum Genet* 22, 1040-5 (2014).
[13] Rieux, A. *et al.* Improved calibration of the human mitochondrial clock using ancient genomes. *Mol Biol Evol* 31, 2780-92 (2014).

Table S2.

SampleID	Laboratory	Range HVS-I	Range HVS-II	Haplogroup	Positions
40-1001	Argentinean lab	16024-16365	73-340	T1a1'3	73G 152C 195C 263G 315.1C 16126C 16163G 16186T 16189C 16239T 16294T
40-1002	Argentinean lab	16024-16365	73-340	D1j	73G 152C 263G 315.1C 16223T 16242T 16311C 16325C
40-1004	Argentinean lab	16024-16365	73-340	U5b3b1	73G 150T 189G 228A 263G 309.1C 315.1C 16192T 16270T 16304C
40-1006	Argentinean lab	16024-16365	73-340	T	73G 152C 263G 309.1C 315.1C 16126C 16294T 16304C
40-1007	Argentinean lab	16024-16365	73-340	D1j	73G 152C 263G 309.1C 315.1C 16172C 16223T 16242T 16311C 16325C 16362C
40-1008	Argentinean lab	16024-16365	73-340	A2a	73G 146C 153G 235G 263G 309.1CC 315.1C 16111T 16192Y 16223T 16290T 16319A 16362C
40-1017	Argentinean lab	16024-16365	73-340	T1a1'3	73G 152C 195C 263G 309.1C 315.1C 16126C 16163G 16186T 16189C 16294T
40-1019	Argentinean lab	16024-16365	73-340	D1	73G 195C 263G 315.1C 16223T 16325C 16362C
40-1031	Argentinean lab	16024-16365	1-576	H1c	263G 309.1CC 315.1C 477C 16519C
40-1035	Argentinean lab	16024-16365	1-576	H1+152	55C 56G 57G 60.1T 71d 152C 263G 315.1C
40-1036	Argentinean lab	16024-16365	1-576	U2e1	73G 152C 263G 315.1C 340T 508G 523d 524d 16051G 16129C 16172C 16183C 16189C 16362C 16519C
AG	Spanish lab	16024-16365	1-576	H1c3	195C 257G 263G 309.1C 315.1C 477C 16519C
ASE	Spanish lab	16024-16365	1-576	H24	263G 309.1CC 315.1C 573.1C 16293G 16519C
